# Effect of Multi-Metal Composition on Arsenic Sorption by LDHs: A Comparative Study of MgCuZnAl-LDH and MgAl-LDH

**DOI:** 10.3390/molecules31152645

**Published:** 2026-07-29

**Authors:** Agnieszka Lipke, Agnieszka Sawicka, Bartosz Płaska, Mariusz Trytek, Grzegorz Wójcik, Diana Vistorskaja, Denis Sokol, Agnieszka Gładysz-Płaska, Marek Majdan, Aivaras Kareiva

**Affiliations:** 1 Institute of Chemistry, Faculty of Chemistry and Geosciences, Vilnius University, Naugarduko Street 24, 03225 Vilnius, Lithuania; diana.vistorskaja@chgf.vu.lt (D.V.); denis.sokol@chgf.vu.lt (D.S.); aivaras.kareiva@chgf.vu.lt (A.K.); 2 Institute of Chemical Sciences, Faculty of Chemistry, Maria Curie-Sklodowska University in Lublin, Maria Curie-Skłodowska Square 2, 20-031 Lublin, Poland; agnieszka.sawicka12@wp.pl (A.S.); grzegorz.wojcik2@mail.umcs.pl (G.W.); agnieszka.gladysz-plaska@mail.umcs.pl (A.G.-P.); majdan.marek8@gmail.com (M.M.); 3 Faculty of Medicine and Dentistry, Medical University of Lublin, Racławickie Avenue 1, 20-059 Lublin, Poland; bartosz.plaska@onet.pl; 4 Institute of Biological Sciences, Faculty of Biology and Biolotechnology, Maria Curie-Sklodowska University in Lublin, Akademicka Street 19, 20-033 Lublin, Poland; mariusz.trytek@mail.umcs.pl

**Keywords:** layered double hydroxides, arsenate(V) adsorption, LDH stability, multimetallic LDH, water treatment

## Abstract

Multimetallic layered double hydroxides (LDHs) are promising oxyanion sorbents, but their practical use requires evaluation of sorption efficiency and chemical stability. This study examined how introducing Cu^2+^ and Zn^2+^ into the LDH structure affects As(V) sorption, comparing binary Mg_3_Al_1_ and four-component Mg_2_Cu_0.5_Zn_0.5_Al_1_ in carbonate and chloride forms. The materials were synthesised by the co-precipitation method and characterised using XRD, FTIR, SEM, BET, and XPS, while their layer metal composition was determined by ICP-OES. The sorption studies were supplemented by chemical stability analysis (pH 4–11) and As(V) desorption experiments. Equilibrium data were evaluated using the Redlich–Peterson isotherm model. The four-component LDH showed higher As(V) sorption capacity than the conventional MgAl system, with the best performance observed for chloride forms: 37.5 mg/g for MgCuZnAl-LDH and 22.5 mg/g for MgAl-LDH. The sorption kinetics are well described by the pseudo-second-order and Elovich models, indicating a significant contribution from chemisorption. The results suggest that As(V) removal involves the combined action of anion exchange, electrostatic interactions and inner-sphere complex formation, controlled by the composition of the LDH layer and the type of interlayer anion. The introduction of Cu^2+^ and Zn^2+^ promoted additional active sites and stronger As(V) surface interactions. The results indicate that MgCuZnAl-LDH, particularly in the chloride form, exhibits promising As(V) sorption performance under model conditions involving a single solute.

## 1. Introduction

Arsenic is a harmful and carcinogenic element. It occurs naturally in the environment as a result of processes such as soil erosion, weathering, and the dissolution of minerals (e.g., pyrite and arsenopyrite). However, its excessive presence in water systems and soil stems from anthropogenic sources, where it is related to the use of artificial fertilisers and pesticides, as well as emissions from the mining and smelting of ores such as copper, nickel, gold and lead, from coal combustion, and constantly growing steel production [1]. The United States Environmental Protection Agency (USEPA) recognises arsenic as one of the most toxic elements in the world [2]. It ranks second on the 2025 Substance Priority List compiled by the Agency for Toxic Substances and Disease Registry (ATSDR), which prioritises hazardous substances based on their frequency of occurrence at National Priorities List sites, toxicity, and potential for human exposure [3]. In consideration of the above, in 1993, the World Health Organization (WHO) reduced the maximum permissible level of As in drinking water from 0.05 to 0.01 mg/L [1].

Among the methods investigated for arsenic removal from water are coagulation/precipitation, ion exchange, oxidation, phytoremediation, membrane processes and adsorption [4,5]. Among them, adsorption has attracted particular attention because of its operational simplicity, the possibility of using relatively inexpensive solid materials, and the potential for sorbent regeneration [4]. Therefore, a number of materials have been used as adsorbents in studies on arsenic adsorption, including poorly crystallised aluminium oxides, poorly crystallised iron hydroxides, montmorillonite, kaolinite, goethite, gibbsite, hematite, organic matter, silicate clays, soils, activated carbon and layered double hydroxides [1]. The latter, due to their large specific surface area, low cost, ease of synthesis, and reusability, are expected to become a widely used group of promising effective adsorbents [1,6,7].

Layered double hydroxides (LDHs), also known as anionic clays, are a class of layered minerals with exchangeable anions in the interlayer spaces. Their crystalline structure consists of positively charged layers [M^2+^_1−x_M_x_^3+^(OH)_2_]^x+^, similar to the brucite Mg(OH)_2_ structure, and a negative interlayer region containing anions and water molecules, A^m−^_x/m_ nH_2_O. This composition can be expressed by the general formula [M^2+^_1−x_M^3+^_x_(OH)_2_]^x+^A^m−^_x/m_·nH_2_O, where M^2+^ is a divalent metal cation (e.g., Mg, Cu, Zn, Ni, Fe, Co, or Mn), M^3+^ is a trivalent cation (e.g., Al, Fe, Cr, Mn, Sc, Ga, Ni, or Co), and A stands for an anion with interlayer charge m^−^. The value of x must be between 0.17 and 0.33 to ensure LDH stability. An important advantage of layered double hydroxides is the possibility of tailoring their composition by substituting M^2+^ and/or M^3+^ cations, which enables the preparation of binary, ternary, quaternary, and more complex LDHs with different divalent and trivalent metal cation combinations [8,9], and may result in synergistic effects, for example in sorption [10]. In turn, the exchange of interlayer anions, for example with other inorganic anions, organic anions, or anions of coordination compounds, allows modulation of the interlayer spacing. The order of exchangeability of common anions depends on their type and is as follows: CO_3_^2−^   >  SO_4_^2−^, OH^−^   >   F^− ^  >   Cl^−^   >   Br^−^   >   NO_3_^−^ [8,11]. Polyvalent anions are easily inserted into the interlayer space, whilst low-valent anions are readily exchanged [12]. For layered double hydroxides composed of Mg, Al, and CO_3_^2−^, the term ‘hydrotalcite’ is generally reserved. When other metals are present or different interlayer anions are dominant, these materials are commonly referred to in the literature as ‘hydrotalcite-type compounds’ [1]. In addition to sorption-related applications, LDHs and their modified forms have also been used in organic and inorganic catalytic processes [13], electrochemistry [14], photochemistry [15], water splitting [16], and oil transesterification for the production of biofuel [17], as well as in water purification from inorganic contaminants and selected organic pollutants [7,18,19,20,21,22].

The pH of the medium is a key factor in studies on the adsorptive removal of environmental pollutants. Layered double hydroxides are alkaline materials, and the pH of the solution strongly affects their behaviour and stability. At low pH, LDHs are eutectic, and their structures can change. Thus, both the easy dissolution of LDHs in acidic solutions and the ease of hydrolysis and polymerisation of heavy metal ions in an alkaline environment negatively affect their removal capacity. Therefore, the optimal pH range for the removal of heavy metal ions using LDHs is 6–8 [8]. An important characteristic of arsenic related to the issue of pH is that its toxicity varies with the chemical species and oxidation state [1]. At the same time, arsenic-bearing wastewaters exhibit a wide pH range depending on their origin (Table 1). Acid mine drainage and some metallurgical effluents are usually strongly acidic (pH 1–5), while other industrial streams, especially after neutralisation, may be close to neutral or even alkaline (pH 7–12). In these systems, arsenic is often present as a mixture of As(III) and As(V), although some wastewaters contain mainly As(V). The form of As(V) in solution depends strongly on pH—under most environmental conditions it occurs mainly as H_2_AsO_4_^−^ and HAsO_4_^2−^.

In a practical context, one of the most important considerations regarding contaminant adsorption, in relation to the pH of the environment, is adsorbent stability [20]. Very few studies have assessed this for hydrotalcites during adsorption and regeneration, even though changes in pH can significantly affect the stability of the layered structure [32]. If LDH precursor metals in brucite-like layers are released into solution, their concentrations should be kept below levels harmful to the environment [1]. This issue is particularly important for LDH materials containing transition metals, as their leaching may affect the environmental safety and practical suitability of the sorbent. According to the WHO Guidelines for Drinking-water Quality [33], copper has a health-based guideline value of 2 mg/L, whereas no formal health-based guideline values are proposed for magnesium, zinc, or aluminium in drinking water. For Al, the WHO indicates practicable levels of 0.1 mg/L or less in large treatment facilities and 0.2 mg/L or less in small facilities; for Zn, concentrations above 3 mg/L may impair consumer acceptability.

The aim of this study was to evaluate the efficiency of As(V) removal, to identify the adsorption mechanism as a function of the adsorbent’s composition, and to determine the stability of the LDH materials under investigation: Mg_3_Al_1_ and Mg_2_Cu_0.5_Zn_0.5_Al_1_. The experiments were carried out in model single-component systems. The scope of the As(V) adsorption studies was supplemented by an analysis of the pH-dependent leaching of structural cations from the materials under investigation.

## 2. Results and Discussion

### 2.1. Characteristics of Sorbents

Mg_3_Al_1_ hydrotalcite and its modified form, in which part of the Mg^2+^ cations was replaced by Cu^2+^ and Zn^2+^ ions, were synthesised by the co-precipitation method, yielding Mg_3_Al_1_-CO_3_ and Mg_2_Cu_0.5_Zn_0.5_Al_1_-CO_3_, respectively. In both cases, the M^2+^/M^3+^ molar ratio was 3. The carbonate forms of these materials (LDH-CO3) were subsequently converted into chloride-containing ones (LDH-Cl) by anion exchange, yielding Mg_3_Al_1_-Cl and Mg_2_Cu_0.5_Zn_0.5_Al_1_-Cl, respectively. All four layered double hydroxide materials were subjected to physicochemical characterisation. For simplicity, in the remainder of this article these sorbents will be designated as MgAl-CO3, MgAl-Cl, MgCuZnAl-CO3, and MgCuZnAl-Cl for the binary Mg_3_Al_1_ and multimetallic Mg_2_Cu_0.5_Zn_0.5_Al_1_ LDHs in the carbonate and chloride forms, respectively.

**Figure 1:**
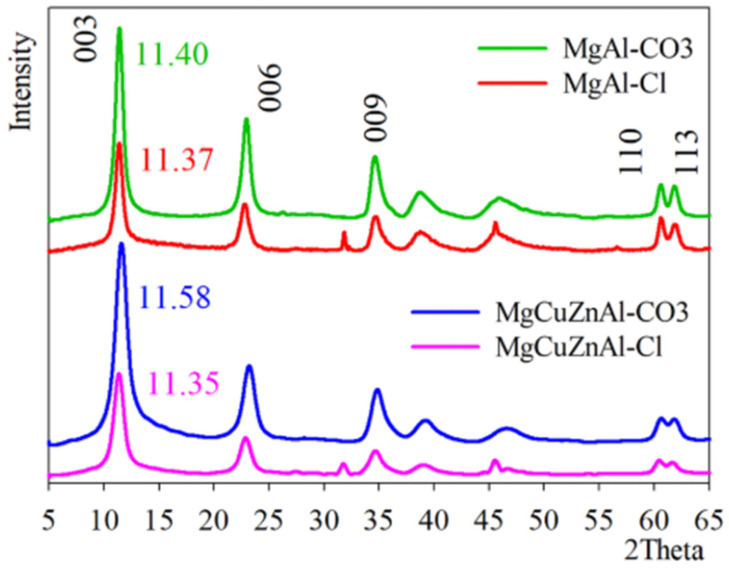
Powder XRD patterns of MgAl and MgCuZnAl in carbonate and chloride forms.

The typical X-ray diffraction (XRD) patterns of the MgAl and MgCuZnAl LDHs as prepared and after carbonate-to-chloride ion exchange are presented in Figure 1. The basal diffraction peaks (003), (006) and (009) and the high-angle reflections (110) and (113) are in good agreement with the characteristics of LDH-type materials reported in the literature [17,34]. The additional peaks at approximately 31.8° and 45.6° originate from residual NaCl used for the anion exchange. As follows from the comparison of MgAl and MgCuZnAl, distinct broadening of the (003) reflection and weaker separation of (110) and (113) are found (Table 2). This may be related to the presence of Cu^2+^ and the resulting local layer distortions, described in the literature as the Jahn–Teller effect, which causes a reduction in the degree of crystalline order in the four-component material [35]. This is also likely related to a more varied cation occupancy within the layers, which promotes local structural disturbances, even though the lattice parameter a, reflecting the average distance between cations in the layer, remains essentially unchanged (Table 2) [9,10]. The lower crystallinity of the multimetallic LDH may also be related to the more complex co-precipitation behaviour of multiple cations appearing in parallel. Differences in the cation speciation, ionic size, and hydroxide solubility can influence nucleation and crystal growth during the LDH formation. After the carbonate–chloride ion exchange, a significant increase is observed in both lattice parameters a (3.054–3.062 Å) and c (22.92–23.40 Å) as well as d(003) (7.64–7.80 Å). This suggests partial replacement of carbonate by the more weakly bound and more largely hydrated chloride species in MgCuZnAl. This results in structural loosening and an associated increase in interlayer spacing. The described changes are not as pronounced for MgAl, where the exceptionally stable carbonate structure allows for only a slight exchange of anions. However, upon contact with NaCl, this results mainly in enhanced chloride adsorption on the matrix surface [36]. Such reduced crystallinity is not necessarily disadvantageous, since it may increase the density of structurally diverse and potentially reactive sites.

Based on the ICP-OES analysis and following normalisation of the obtained composition to Al = 1, the actual structure of the cationic layer in synthesised LDH-CO3 was determined. Both materials retained the layered double hydroxide framework. For the sample with the assumed theoretical composition [Mg_2_Cu_0.5_Zn_0.5_Al_1_(OH)_8_]^+^, the formula [Mg_1.88_Cu_0.51_Zn_0.50_Al_1_(OH)_7.78_]^+^ was obtained, indicating a very good agreement. For [Mg_3_Al_1_(OH)_8_]^+^, it was calculated that Mg/Al = 2.76, yielding [Mg_2.76_Al_1_(OH)_7.52_]^+^. The observed Mg^2+^ deficiency relative to the theoretical composition, approximately 6% and 8%, respectively, should be evaluated cautiously, since the ICP-OES analysis of solid samples involves digestion, dilution, and matrix-dependent calibration. Consequently, minor deviations may be partly attributable to sample preparation and analytical uncertainty, rather than solely to actual structural changes. However, these small differences between the nominal composition of the solution and that of the final precipitate are possible in LDHs obtained by the co-precipitation method. This is because the process depends on the synthesis conditions, the type of precursors, the precipitating agent, the pH, and the simultaneous precipitation of divalent and trivalent metal hydroxides [37,38]. At the same time, a very good agreement is observed between the experimental Cu/Al and Zn/Al ratios and the theoretical values, which confirms preservation of the assumed cationic structure of LDH (Figure 1). Following the anion exchange process to chloride forms, a systematic decrease in the M^2+^/Al ratio was observed. For the tetrametallic LDH-Cl, the composition of the brucite-like layer was [Mg_1.72_Cu_0.47_Zn_0.49_Al_1_(OH)_7.36_]^+^. Compared to MgCuZnAl-CO3, the Mg/Al ratio decreased more significantly, from 1.88 to 1.72. A similar effect was observed for MgAl-Cl, where Mg/Al dropped from 2.76 to 2.63, which corresponds to the layer of [Mg_2.63_Al_1_(OH)_7.26_]^+^. These changes indicate that the acid-assisted CO_3_^2−^/Cl^−^ exchange is not an entirely neutral preparative step and is not limited solely to the interlayer space, but it can also affect the composition of the cationic layer slightly. Therefore, it may induce partial restructuring of the crystallite surfaces or edges, possibly accompanied by slight dissolution of the most susceptible layer fragments. This is comprehensible, as carbonate anions are particularly strongly bound within the LDH network [11,39,40].

Porosity of the synthesised LDH sorbents was assessed using nitrogen adsorption/desorption porosimetry. The results showing the BET specific surface area, the micropore area, as well as the mesopore volume and average pore size, determined on the basis of BJH analysis, are presented in Table 3. The average pore diameter values of all LDH materials vary in the range 12–30 nm, being within the mesoporous range. This is further supported by the N_2_ adsorption–desorption isotherms presented in the Supplementary Information (Figures S1 and S2, Table S1), which show type IV behaviour and visible hysteresis loops, confirming the mesoporous character of the LDH materials. Based on the hysteresis-loop behaviour, MgAl samples consistently exhibited a larger relative hysteresis gap and a lower loop-closure point than MgCuZnAl samples, indicating systematic differences in pore geometry and connectivity between the two LDH systems. These features suggest more pronounced H2-like hysteresis behaviour for the binary MgAl materials, which may be associated with an ink-bottle-type pore effect. The presence of Cu^2+^ and Zn^2+^ in the quaternary MgCuZnAl materials appears to favour a more open and better-connected mesoporous network, with hysteresis behaviour closer to H3-type loops, typically associated with slit-shaped interparticle pores formed between loosely aggregated LDH platelets. The BET surface area obtained for MgAl-CO3 and MgCuZnAl-CO3 is consistent with the values reported in the literature [41,42,43]. The large specific surface area of MgAl (103 m^2^/g) can be attributed to the fact that, in terms of its interlayer structure, MgAl-CO3 is commonly considered to exist in a well-ordered and stable hydrotalcite-like phase. The CO_3_^2−^ anion favours the preservation of a well-developed LDH structure, which may contribute to more efficient surface accessibility to N_2_ during porosimetry [44]. The smaller BET surface area for MgCuZnAl-CO3 (90 m^2^/g), as well as the increased pore volume and diameter, are related to the introduction of Cu^2+^ and Zn^2+^ ions. In line with the conclusions from the XRD studies, this may suggest a change in crystallinity and in morphology towards a more compact arrangement of the plates, thereby reducing the accessible surface area [9]. The CO_3_^2−^ → Cl^−^ anion exchange process in both matrices resulted in a reduction in the BET surface area and pore volume. For MgAl, the particularly large decrease in the BET surface area (BET 103 → 20 m^2^/g) may be related to the dominant contribution of surface chloride adsorption reported for the Mg–Al hydrotalcite-type phases [36]. This could reduce the accessibility of part of the surface to nitrogen during porosimetry. The smaller decrease observed for MgCuZnAl may be related to a different structural reorganisation of the multimetallic LDH during chloride treatment. It should be noted that the surface parameters are largely dependent on the conditions of LDH synthesis, washing, and drying [45]. Therefore, no universal trend in the BET surface area as a function of the interlayer anion type (e.g., CO_3_^2−^ vs. Cl^−^) has been established in the literature.

**Figure 2:**
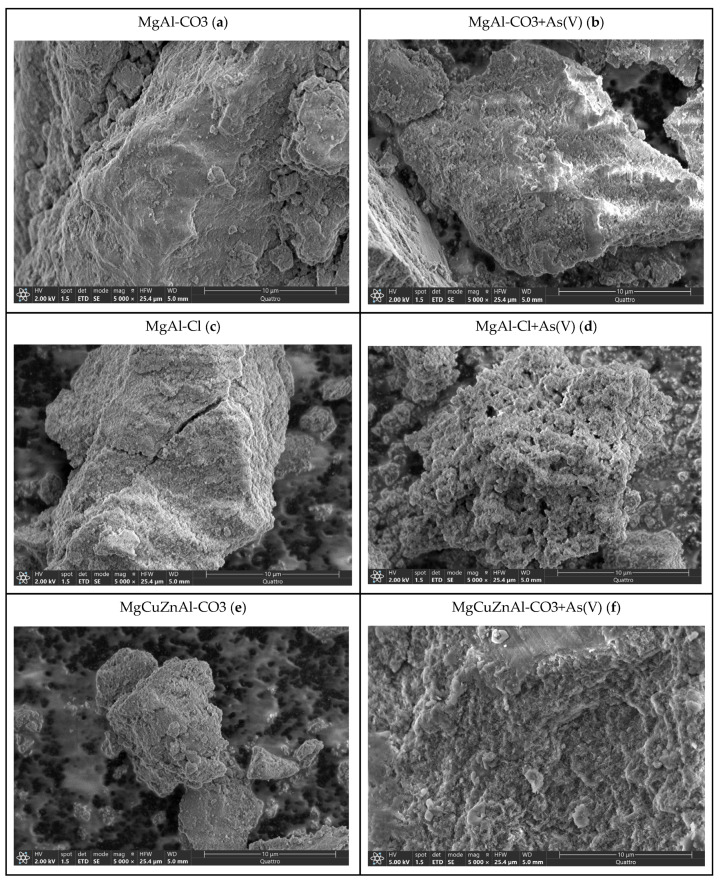

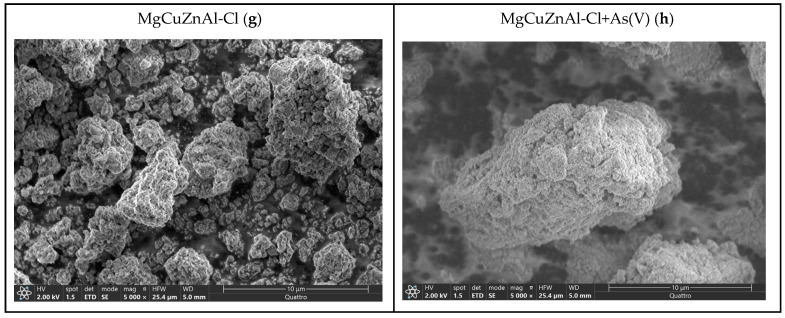
SEM images of MgAl and MgCuZnAl in the carbonate and chloride forms before (**a**–**d**) and after (**e**–**h**) As(V) adsorption.

SEM images (Figure 2) illustrate the surface morphology of the synthesised LDH samples before (a, c, e, g) and after (b, d, f, h) As(V) adsorption. MgAl-CO3 (a) exhibits the most ordered morphology: relatively smooth, broad plate-like aggregates with a more compact, although still lamellar, structure are visible. MgAl-Cl (c) is clearly more condensed and smoothed, with fewer open inter-aggregate spaces, which corresponds well to the earlier sharp decrease in the BET surface area following the CO_3_^2−^ → Cl^−^ exchange. This more compact morphology may partly explain why the chloride form of MgAl did not show such a clear improvement in As(V) adsorption as the MgCuZnAl-Cl material, despite the presence of more easily exchangeable chloride ions. In the case of the four-component material, both MgCuZnAl-CO3 (e) and MgCuZnAl-Cl (g) have a more heterogeneous, rough, and less regular texture than the MgAl samples. MgCuZnAl-CO3 still retains some of the characteristics of plate-like aggregates, whereas MgCuZnAl-Cl forms finer, more irregular agglomerates. Such a less regular morphology is consistent with the higher As(V) uptake observed for the MgCuZnAl samples, as it may provide more accessible external and edge sites for arsenate binding. These observations are consistent with the earlier XRD and BET analysis: the MgAl-CO3 form was the most ordered, MgAl-Cl was the most ‘closed’ in terms of texture after chlorination, and the four-component materials were more heterogeneous even before the sorption.

### 2.2. Sorption Studies

#### 2.2.1. Kinetics

**Figure 3:**
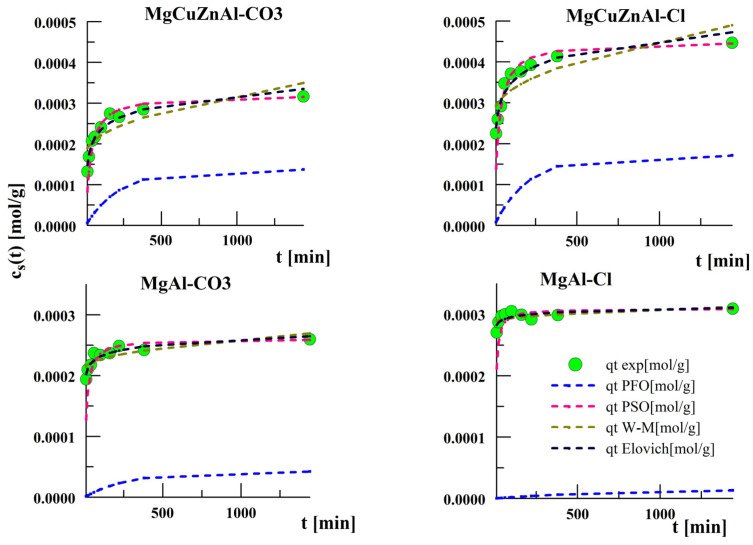
The change in As(V) concentration in sorbent phase with time (As(V) initial concentration c_in_ = 0.0005 mol/L, aqueous phase—0.025 L, sorbent phase—0.025 g, temperature—298 K).

Figure 3 shows the change in arsenate sorption, c_s_(t), over time based on various kinetic models: pseudo-first-order (PFO), pseudo-second-order (PSO), Weber–Morris (W-M) and Elovich. Detailed data on the kinetic parameters are given in Table 4.

##### Pseudo-First-Order (PFO) Model

The pseudo-first-order (PFO) model, proposed by Lagergren in 1898 [46], describes the rate of adsorption as being proportional to the difference between the adsorption capacity at equilibrium and the amount of metal currently adsorbed [47]:

dq_t_/dt = k_1_ (q_e_ − q_t_), (1)

where q_t_ (mg/g or mol/g) is the amount of metal adsorbed at time t; q_e_ (mg/g or mol/g) is the adsorption capacity at equilibrium; k_1_ (1/min) is the first-order adsorption rate constant; and t (min) is contact time. After integrating with the initial condition q_t_ = 0 for t = 0, the following integral form is obtained:

(2) $$q_{t}=q_{e}\cdot \left(1-e^{-k_{1}\cdot t}\right)$$

The linear form historically used to determine parameters by linear regression is:

ln(q_e_ − q_t_) = ln q_e_ − k_1_ · t (3)

A plot of ln(q_e_ − q_t_) against time t yields a straight line with a slope of −k_1_ and a y-intercept of ln q_e_.

Under our experimental conditions, we did not achieve a state of full equilibrium after 24 h. The sorption of arsenates on all the LDH materials studied did not reach a clear equilibrium plateau within the time interval under investigation. However, the sorption rate decreased markedly over time: for the MgAl samples, the increase in c_s_(t) between t = 380 min and t = 1440 min was less than 8%, while for the MgCuZnAl samples it did not exceed 12%. Therefore, t = 24 h (1440 min) was adopted as the contact time for subsequent isothermal experiments, corresponding to a state of pseudo-equilibrium that ensured comparable and reproducible sorption conditions for all the materials under investigation.

Ultimately, therefore, we relied on the equation:

ln(c_s_ − c_s_(t)) = ln c_s_ − k_1_ · t, (4)

where c_s_ refers to the concentration of As(V) anions in the sorbent phase after 24 h.

Although the linearised form of the PFO model yielded acceptable R^2^ values (Table 4), the calculated equilibrium sorption capacities (c_s calc_) were severely underestimated compared with experimental values, indicating that the model fails to describe the sorption process adequately, a known artefact of the linearisation procedure.

##### Pseudo-Second-Order (PSO) Model

Based on the work of Ho and McKay, the following differential equation was used [47]:

dc_s_/dt = k_2_ · (c_s_ − c_s_(t))^2^, (5)

which, upon integration, takes the following form:

c_s_(t) = k_2_ · c_s_^2^ · t/(1 + k_2_ · c_s_· t). (6)

Upon linearization, the equation takes the following form:

t/c_s_(t) = 1/(k_2_ · c_s_^2^) + (1/c_s_) · t. (7)

A plot of t/c_s_(t) vs. t yields a straight line with a slope of (1/c_s_) and an intercept of (1/(k_2_ · c_s_^2^)). The pseudo-second-order model provided the best fit to the kinetic data (R^2^ > 0.999), indicating that the sorption rate may be governed by specific interactions at the sorbent surface, including anion exchange and/or surface complexation, as reported for analogous LDH systems [47].

The values of the rate constant k_2_ form the following series: MgAl-Cl ≫ MgAl-CO3 > MgCuZnAl-CO3 ≈ MgCuZnAl-Cl; thus, whilst the k_2_ values for two-component LDHs are higher than those for four-component LDHs, the c_s_(t) values obtained are lower, which indicates a slow but more efficient sorption process for MgCuZnAl-CO3 and MgCuZnAl-Cl, probably associated with the formation of inner-sphere complexes of HAsO_4_^2−^ ions with Cu^2+^ ions. A comparison of the kinetics of Cl^−^/HAsO_4_^2−^ ion exchange with that of CO_3_^2−^/HAsO_4_^2−^ favours chlorides, but only clearly in the case of the two-component LDH, which indirectly suggests that, in the case of the four-component LDH, ion exchange is not the sole mechanism determining the sorption of As(V) anions.

##### The Weber–Morris (W-M) Model

The equation describing the sorption of As(V) in the Weber–Morris (W-M) model is as follows [48]:

c_s_(t) = k_id_ · √t + C, (8)

where k_id_ (mol/(g·√min)) is the intraparticle diffusion constant; and C (mol/g) is the intercept, which is a measure of the external resistance of the boundary layer.

If C = 0, intraparticle diffusion governs the entire process. If C > 0 (as in the present data), intraparticle diffusion is not the sole rate-limiting step. For all sorbents, R^2^ = 0.38–0.70 when C > 0, which unequivocally rules out intraparticle diffusion as the controlling step.

##### Elovich’s Model

Based on the work of Chien and Clayton [49] we have adopted the following equation:

c_s_(t) = (1/β) · ln(α · β · t + 1), (9)

which, in its linearised form, takes the simplified form (α · β · t ≫ 1):

c_s_(t) = (1/β) · ln(α · β) + (1/β) · ln(t) (10)

where α (mol/(g·min)) is the initial sorption rate, and β (g/mol) is the Elovich model parameter determining the rate at which the sorption rate decreases as surface loading increases. The c_s_(t) vs. ln(t) plot should be linear and describes systems with heterogeneous sorption sites, which is more evident in the case of both forms of four-component LDH, which achieve R^2^ values above 0.9 compared with two-component LDH.

The sorption of As(V) ions on LDH materials may be governed by the following interacting mechanisms:

Mechanism 1—formation of inner-sphere complexes with Cu^2+^ (the most likely mechanism for both forms of MgCuZnAl). The HAsO_4_^2−^ ions can form complexes with Cu^2+^ ions located at the edges of the LDH brucite layers, displacing OH^−^ groups. The Jahn–Teller effect causes distortion of the [Cu(OH)_6_] octahedra. The bonds at the equatorial sites are shorter and more reactive, which may facilitate ligand exchange. This mechanism explains both the higher (c_s_) values obtained for MgCuZnAl and the lower rate constant (k_2_) values compared with the MgAl materials. This is because the binding of arsenates to Cu^2+^ centres is stronger, but requires a longer time for the coordination sphere to reorganise. This mechanism may be supported by the Elovich model, perhaps even more convincingly than by the pseudo-second-order kinetic model. The values of the parameter (α), which determines the initial sorption rate, differ by several orders of magnitude between sorbents. The parameter (α) reaches significantly higher values for MgAl-Cl (α ≈ 2.13 × 10^15^ mol/(g·min)) and MgAl-CO3 (α ≈ 12.4 mol/(g·min)) than for four-component materials, for which its values are of the order of (10^−4^) mol/(g·min). Such an extreme value of α for MgAl-Cl, coupled with a low R^2^ = 0.583, suggests that the Elovich model is not suitable for this material. The MgAl-Cl is characterised by kinetics dominated by a single anion exchange (Cl^−^ → HAsO_4_^2−^), which is similar to a homogeneous process, for which the Elovich model is not a suitable tool. The parameter β, which determines the rate at which the sorption rate decreases as surface loading increases, takes on the highest values for MgAl (β ≈ 7.95 × 10^4^ and 1.76 × 10^5^ g/mol) compared with Cu/Zn materials (β ≈ 2.13–2.66 × 10^4^ g/mol). Lower β values for MgCuZnAl indicate a slower decrease in the sorption rate as coverage increases, which suggests a time-extended process of coordination reorganisation involving Cu^2+^.

Mechanism 2—anion exchange in the interlayer space (occurs in both sorbents: MgCuZnAl and MgAl) according to the schematic equation:

2[LDH–Cl] + HAsO_4_^2−^ → [(LDH)_2_–HAsO_4_] + 2Cl^−^, (11)

and the higher k_2_ value for Cl^−^ compared to CO_3_^2−^ explains the easier exchange of Cl^−^ for HAsO_4_^2−^. This mechanism dominates in the MgAl, where the absence of Cu^2+^ eliminates coordination complexation.

Mechanism 3—inner-sphere complexes with Zn^2+^.

The Zn^2+^ ion, with a d^10^ configuration, does not exhibit the Jahn–Teller effect, ensuring the symmetry of the octahedra. This ion’s affinity for arsenates is lower than that of Cu^2+^, but at the same time higher than that of Mg^2+^; hence, mechanism 3 may interact with mechanism 1.

#### 2.2.2. Adsorption Isotherms

**Figure 4:**
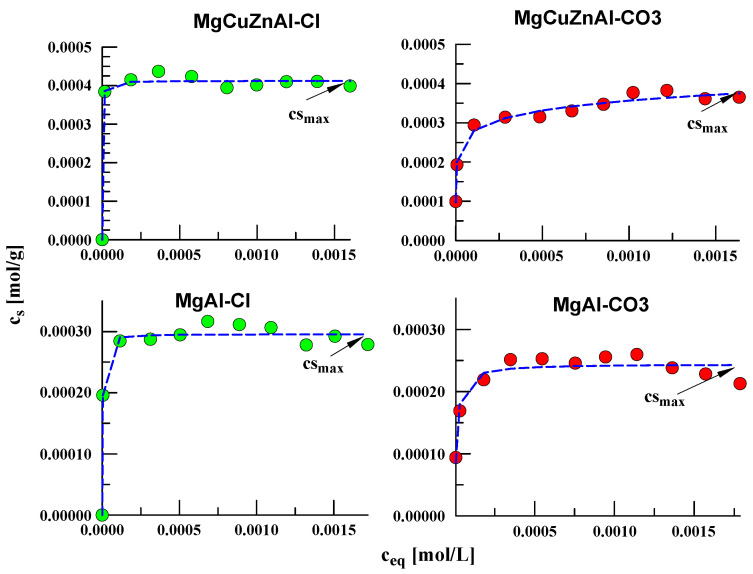
Adsorption isotherms for As(V) ions on LDH materials (dashed line—fit).

Figure 4 shows the adsorption isotherms for As(V) ions on the LDH materials, whereas Table 5 summarises the determined sorption parameters. The data were analysed using the Redlich–Peterson equation [50]. This model describes the adsorption equilibrium on heterogeneous surfaces and combines features of the Langmuir and Freundlich isotherms:

c_s_ = (K_RP_ · c_eq_^β^)/(1 + a_RP_ c_eq_^β^), (12)

where c_s_ (mol/g) is the concentration of As(V) in the sorbent phase after 24 h of contact; c_eq_ (mol/L) is the equilibrium concentration of As(V) in the aqueous equilibrium phase; K_RP_ (L/g) is the R-P constant, a measure of affinity at low concentrations, within the linear range of the isotherm; a_RP_ ((L/mol) ^β^) is the R-P constant describing sorption capacity and non-linearity; and β (-) is the heterogeneity exponent: β = 1 reduces the R-P model to the Langmuir model, 0 < β < 1 describes a heterogeneous surface.

The point (0, 0) was also included when fitting the model to the data obtained for the chloride and carbonate forms of MgCuZnAl. This represents a situation in which there is no As(V) in the solution; consequently, the amount of As(V) bound by the sorbent is also zero. This point does not originate from an experimental measurement, but was adopted as a natural boundary condition resulting from the nature of the sorption process. The experimental data covered concentrations greater than zero. At the lowest concentrations used, complete, i.e., 100%, removal of As(V) from the solution was observed. Including the (0, 0) point therefore allowed for a better description of the initial course of the isotherm and ensured the consistency of the model with the physical course of the process. However, this should not be treated as an additional experimental result, but solely as a boundary condition of the model. The maximum adsorption capacity values, derived from the predictions of the Redlich–Peterson model, formed the following series: MgCuZnAl-Cl (0.41 mmol/g, 30.72 mg/g) > MgCuZnAl-CO3 (0.37 mmol/g, 27.72 mg/g) > MgAl-Cl (0.30 mmol/g, 22.48 mg/g) > MgAl-CO3 (0.24 mmol/g, 17.98 mg/g). The initial pH (pH_in_) of the arsenate(V) solution series ranged from 7.2 to 8.1. After 24 h of sorption, the pH (pH_24h_) ranged from 7.9 to 8.1 for MgAl-CO3, 7.6 to 7.8 for MgAl-Cl, 7.5 to 7.6 for MgCuZnAl-CO3, and 6.3 to 7.2 for MgCuZnAl-Cl. The results obtained indicate, first and foremost, the greater effectiveness of the four-component MgCuZnAl materials compared with the two-component MgAl materials. This demonstrates their greater affinity for As(V) ions. This effect can be attributed to the partial substitution of Mg^2+^ ions by Cu^2+^ and Zn^2+^ ions, leading to a distortion of the LDH crystal lattice, an increase in specific surface area and, consequently, an increase in the number of available active sites. For both types of LDH—tetracomponent and bicomponent—the chloride forms exhibited higher c_s max_ values than their corresponding carbonate forms. This difference stems from the properties of the anions present in the interlayer spaces. The CO_3_^2−^ ion, due to its high charge density, is strongly bound to the LDH structure and is difficult to exchange. In contrast, the monovalent Cl^−^ ion is bound more weakly and can therefore be more easily displaced by As(V) anions. The values of the heterogeneity coefficient β indicate the quasi-heterogeneous nature of the surfaces of the MgCuZnAl-Cl, MgAl-Cl and MgAl-CO3 materials, and the distinctly heterogeneous nature of the MgCuZnAl-CO3 surface. A quasi-heterogeneous surface is characterised by the presence of a large number of strong sorption sites with similar energies. This does not rule out the presence of weaker active centres; however, their contribution may not be clearly discernible using the model employed. The high K_RP_ value obtained for MgCuZnAl-CO3, as with MgAl-CO3, can indicate strong surface adsorption occurring at low concentrations of As(V), rather than anion exchange in the interlayer spaces. This process can occur primarily at the edges of crystallites or involve surface hydroxyl groups. However, the number of such sites is limited, which is why they quickly become saturated. Chloride forms, on the other hand, offer an additional mechanism involving anion exchange in the interlayer spaces. This mechanism becomes significant at higher concentrations of As(V) and leads to a higher final sorption capacity, even despite lower K_RP_ values.

The sorption experiments in this study were carried out in single-component Na_2_HAsO_4_ solutions. Consequently, the results obtained represent a model system designed to compare the intrinsic sorption affinity of As(V) for the LDH materials under investigation and to identify the main factors controlling this process. In real environmental and industrial waters, arsenates usually co-exist with other inorganic anions, including sulphate, phosphate, bicarbonate, silicate and chloride. These ions may compete with As(V) for positively charged surface sites and interlayer exchange sites, thereby reducing sorption efficiency. This effect may be particularly significant in the case of phosphates and carbonates/bicarbonates due to their strong affinity for LDH structures. Consequently, these results should be regarded as a baseline assessment, whilst further studies using multicomponent solutions, synthetic groundwater or selected industrial effluents are necessary to verify the suitability of MgCuZnAl-LDH materials under realistic conditions.

#### 2.2.3. Effect of pH and Temperature on As(V) Adsorption

**Figure 5:**
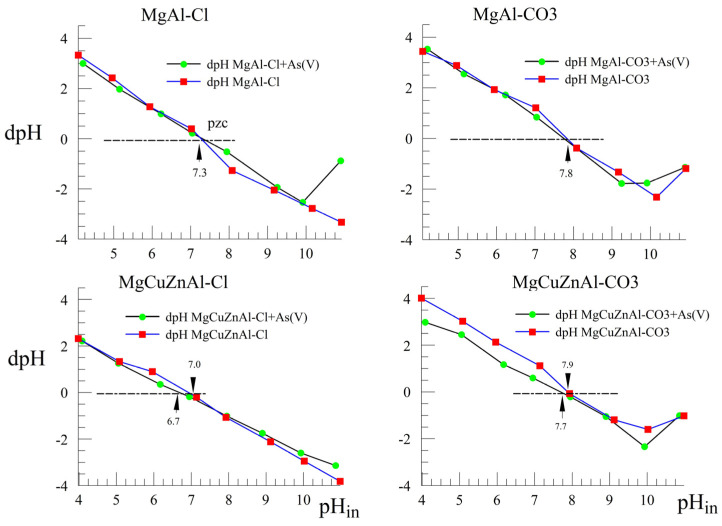
The changes in dpH during sorption of As(V) anions on MgAl and MgCuZnAl in chloride and carbonate forms (comparison with the system without arsenates).

Figure 5 shows the change in pH value of the aqueous phase after adsorption of As(V) anions on MgAl(-Cl or -CO3) and MgCuZnAl(-Cl or -CO3). The change in pH was defined as:

dpH = pH_24h_ − pH_in_ (13)

where pH_in_ is the initial pH of the As(V) solution and pH_24h_ is the pH of the solution after a 24 h sorption process. The zero charge point (pzc) is the pH at which dpH = 0, i.e., at which the surface charge is zero. The surface charge, ranging from positive through zero to negative, results from a change in ≡S–OH_2_^+^ → ≡S–OH → ≡S–O^−^, i.e., changes in the protonated form of the sorbent surface S to a neutral form, and finally to a negative form as the pH of the solution increases. This means that the surface takes on a positive charge to the left of the pzc point (for lower pH values) and a negative charge to the right of the pzc (higher pH values). This results in the sorbent surface becoming negatively charged at pH values above the zero charge point, which means that As(V) ions (with a negative charge) exhibit decreasing affinity to the surface as the pH increases, due to electrostatic repulsion. The adsorption of As(V) ions on the sorbent surface gives it a negative charge, which facilitates surface deprotonation, i.e., transition ≡S–OH_2_^+^ → ≡S–OH with an increase in the pH of the solution, and thus the pH of the pzc point shifts towards lower pH values compared to the pzc for the pure sorbent. This is visible on both forms of the MgCuZnAl sorbent. The As(V) anions may facilitate surface deprotonation and may form stronger complexes with surface-exposed Cu(II) and Zn(II) sites. This effect of lowering pzc after As(V) sorption is not observed in the case of the MgAl sorbent, where the dpH values for the pure sorbent and its form with adsorbed As(V) are identical. This is due to the fact that arsenate is attracted to positively charged sites such as the edges of the layers and the protonated ≡Al/Mg–OH_2_^+^ group. This is a purely electrostatic binding—without ligand exchange and without proton release/absorption. EXAFS studies on analogous Mg/Al LDHs (e.g., with NO_3_^−^) clearly show no evidence of specific adsorption—only Coulombic attraction to positively charged surface sites. There is no As–O–Al/Mg chemical bond, so it does not affect the equilibrium constants of protonation/deprotonation of OH groups at the edges [51].

**Figure 6:**
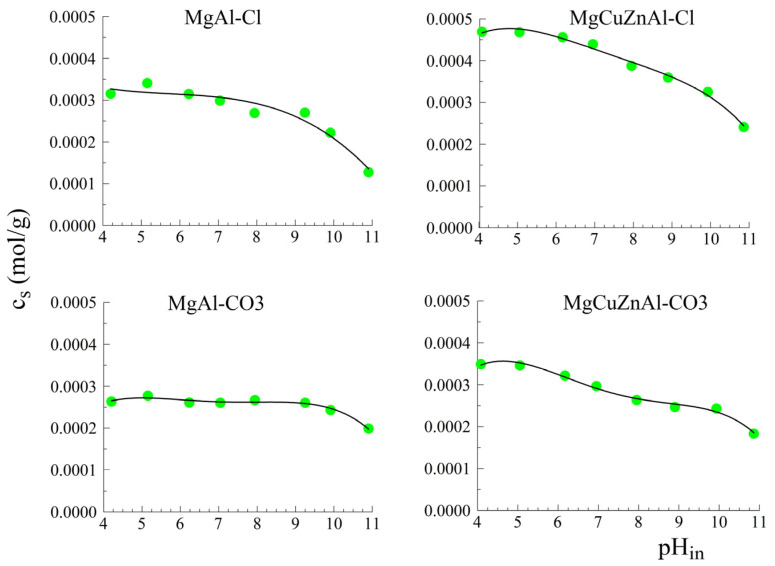
Effect of initial solution pH (pH_in_) on As(V) sorption by MgAl-Cl, MgAl-CO3, MgCuZnAl-Cl and MgCuZnAl-CO3 samples.

Figure 6 shows the effect of pH_in_ on the sorption of As(V) by MgAl and MgCuZnAl. In general, a decrease in As(V) sorption is observed with increasing pH_in_ for both forms of LDH, chloride and carbonate, which is primarily due to strong competition between OH^−^ ions and As(V) ions for sorption sites on the surface and within the LDH structure. In the case of the MgAl, it is clearly evident that the decrease in As(V) sorption is more pronounced for the MgAl-Cl form than for the MgAl-CO3 form, which results from the high affinity of CO_3_^2−^ ions for LDH, and hence their hindered desorption by OH^−^ ions. In the case of MgCuZnAl, this effect is less pronounced, which is likely due to the greater flexibility of the sorbent structure containing Cu and Zn, and consequently the easier penetration by OH^−^ ions.

**Figure 7:**
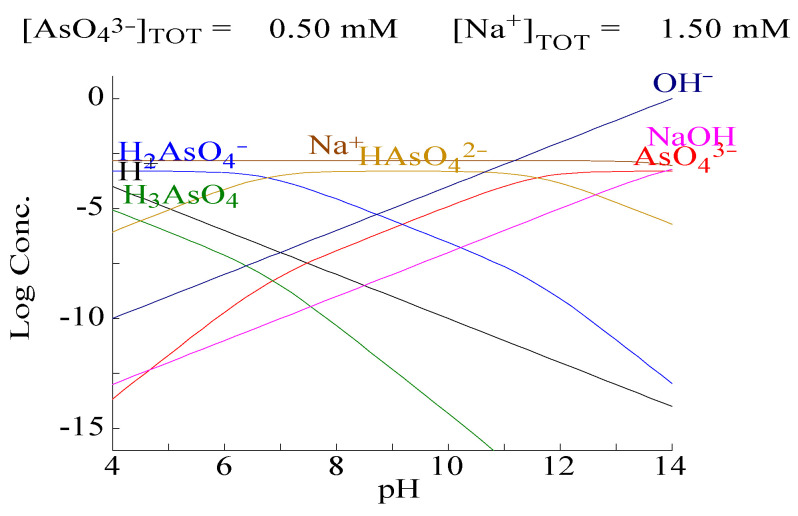
Speciation diagram of As(V) species in Na_3_AsO_4_ solution (total As(V) concentration = 0.5 mmol/L, T = 298 K).

The types of As(V) species present in the solution are best illustrated in Figure 7. In the pH range of 4–11, the main species present are H_2_AsO_4_^−^, HAsO_4_^2−^, AsO_4_^3−^ and, to a lesser extent, H_3_AsO_4_. As the pH increases, the proportions of the individual arsenate forms change, which affects both the value of the partition constant K_d_ and the change in the dpH value. For this reason, the sum logK_d_ + dpH can be considered a linear function of the concentrations of the individual As(V) forms in the initial solution, in accordance with the equation:

(14) $$logK_{d}dpH=a\cdot logc_{{AsO}_{4}^{3-}}+b\cdot logc_{{HAsO}_{4}^{2-}}+c\cdot logc_{{H_{2}AsO}_{4}^{-}}+d\cdot logc_{H_{3}AsO_{4}}+e\cdot {pH}_{in},$$

where the dependent variable logK_d_ dpH denotes the sum of logK_d_ + dpH.

**Figure 8:**
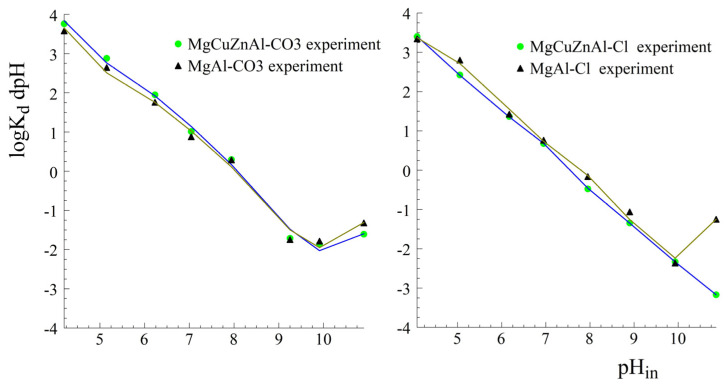
Effect of initial pH (pH_in_) on logK_d_ + dpH values for As(V) sorption on MgCuZnAl-Cl/CO3 and MgAl-Cl/CO3 samples (c_in_ = 0.5 mmol/L). Solid lines correspond to the model calculations.

Figure 8 shows the trend in changes in the logK_d_ dpH value as pH_in_ increases. The values of parameters a, b, c, d and e, referred to as influence parameters, are summarised in Table 6. These parameters were determined using the PSI-Plot software (version 5.5; Poly Software International, Inc., Sandy, UT, USA), whilst the concentrations of individual As(V) forms were calculated using the Medusa software [52]. Very good agreement was obtained between the experimental and calculated values for the sum of logK_d_ + dpH.

Analysis of the influence parameters shows that arsenic acid H_3_AsO_4_ plays virtually no role in the sorption of As(V). The influence coefficient d takes on negative values for all the systems studied, which is related to the very small contribution of this form within the pH range studied and the absence of electrostatic interactions between the neutral H_3_AsO_4_ molecule and the sorbent surface. The influence parameter a, corresponding to the AsO_4_^3−^ ion, also takes on negative values. This can be explained by the strong electrostatic repulsion between the trivalent arsenate anion and the negatively charged sorbent surface in the high pH range. The literature indicates that the sorption of AsO_4_^3−^ on LDH-type materials is very limited, with the forms H_2_AsO_4_^−^ and HAsO_4_^2−^ playing a dominant role [20,51,53].

**Figure 9:**
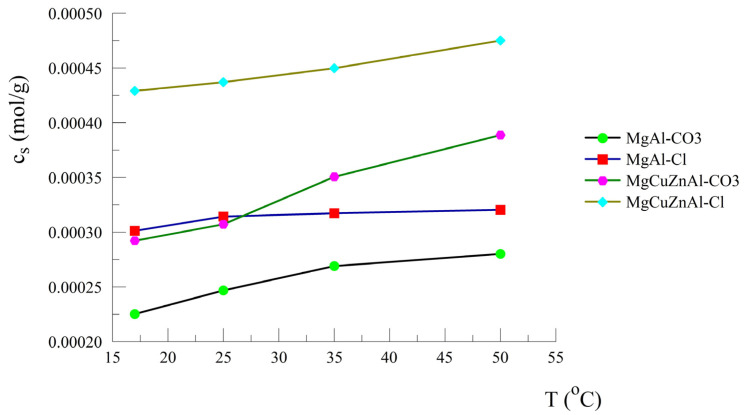
The change in As(V) concentration in sorbent phase with the temperature (As(V) initial concentration 0.5 mmol/L, temperature: 17, 25, 35, 50 °C).

According to the literature, temperature can both increase and decrease the sorption of As(V) on LDH-type materials [20,54,55,56,57]. The composition of the layer (M^2+^/M^3+^), the type of interlayer anion, and whether the material is calcined or uncalcined are of great importance. Figure 9 shows the effect of temperature (17, 25, 35, 50 °C) on the sorption of As(V) on the LDH materials studied. In the case of MgCuZnAl systems, a clear positive effect of temperature on As(V) sorption is observed, whereas for the MgAl materials this effect is much weaker.

The effect of temperature on As(V) sorption on LDH, i.e., changes in standard thermodynamic functions, was evaluated based on the Van’t Hoff equation [58]:

lnK_eq_ = −ΔH°/RT + ΔS°/R, (15)

where K_eq_ is the thermodynamic equilibrium constant of the chemical reaction (in our case K_eq_ = K_d_), ΔH° and ΔS° are the changes in standard enthalpy and entropy, respectively, and R is the gas constant, 8.314 J/(mol K).

The free energy values were calculated from the equation:

ΔG° = −RTlnK_d_ (16)

**Figure 10:**
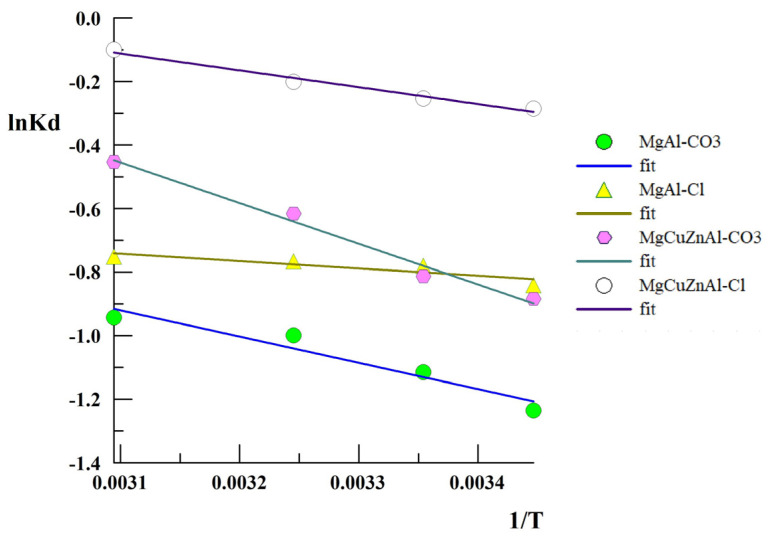
Dependence of lnK_d_ on reciprocal temperature (1/T) for As(V) adsorption on MgAl and MgCuZnAl in carbonate and chloride forms (temperature: 17, 25, 35, 50 °C).

Figure 10 shows the changes in lnK_d_ as a function of the reciprocal temperature, while Table 7 presents the thermodynamic parameters calculated based on the linear fitting parameters of the plots:

lnK_d_ = a(1/T) + b. (17)

All four LDH sorbents showed ΔH° > 0, indicating that As(V) sorption is endothermic, i.e., the adsorption capacity increases with temperature. The endothermic nature of anion adsorption on LDH is a known phenomenon [59,60], and it is driven by the dehydration of both HAsO_4_^2−^ anions and Cu^2+^ cations. In the case of As(V) adsorption on both forms of the MgCuZnAl sorbent, the enthalpy changes were higher than for the MgAl sorbent. The observed difference is clear: MgCuZnAl-CO3 showed ΔH° = 10,651 J/mol compared with 6873 J/mol for MgAl-CO3, corresponding to an increase of approximately 55%; for the LDH–Cl forms, the respective values were 4415 and 1917 J/mol, corresponding to an increase of approximately 130%. An explanation based on the analysed physicochemical data is proposed below.

For MgAl materials, ion exchange of HAsO_4_^2−^ with Cl^−^ or CO_3_^2−^ anions dominates. This is an electrostatic mechanism with a relatively low surface interaction energy. For MgCuZnAl materials, an additional mechanism is likely to accompany ion exchange: the formation of inner-sphere complexes between HAsO_4_^2−^ and Cu^2+^ ions, located at the LDH edges. Inner-sphere As–O–Cu complexes are characterised by higher interaction energy than electrostatic ion exchange, and their formation is more endothermic due to the need for Cu^2+^ dehydration. The formation of these complexes is probably facilitated by the Jahn–Teller effect [61]. Cu^2+^ is a d^9^ ion and is strongly affected by this effect. The octahedral coordination sphere of Cu^2+^ is distorted—two axial Cu–O bonds with water are elongated by approximately 17% compared with the equatorial bonds. This distortion destabilises the octahedral sphere relative to the regular geometry of Mg^2+^ and Zn^2+^ and makes the exchange of axial water molecules for anionic ligands a thermodynamically different process. This effect is most likely significantly responsible for the greater coordination reactivity of Cu^2+^ toward arsenate ions.

The standard hydration enthalpies of the ions (Table 8) show that Cu^2+^ binds water more strongly than Mg^2+^ and Zn^2+^. Therefore, dehydration of Cu^2+^ requires overcoming a higher energy barrier than dehydration of Mg^2+^. More energy must be supplied to the system than in the case of Mg^2+^, which binds water more weakly. This is most likely the reason for the higher ΔH° observed for the MgCuZnAl material. The increase in entropy, ΔS°, for the four-component LDHs (MgCuZnAl-CO3: +29.24 J/mol·K) is clearly higher than for the two-component materials (MgAl-CO3: +13.65 J/mol·K). The higher ΔS° means that adsorption on the MgCuZnAl material probably releases more water molecules into the solution. This is consistent with the proposed mechanism: dehydration of the coordination sphere of Cu^2+^, which has higher |ΔhydH°| value, releases more water molecules than sorption on Mg^2+^ and Zn^2+^.

The equation

ΔG° = ΔH° − TΔS°(18)

indicates that higher ΔH° and higher ΔS° may, at an appropriate temperature, lead to more favourable, i.e., lower, ΔG° values. This is consistent with the observed data: MgCuZnAl-Cl showed the lowest ΔG° among all the investigated materials.

Regardless of the cationic composition of the studied LDHs, the carbonate forms showed higher ΔH° values than the chloride forms, for example MgAl-CO3: 6873 J/mol versus MgAl-Cl: 1917 J/mol. This is consistent with the physicochemistry of anion exchange in LDH: CO_3_^2−^ is an anion with high charge density, forming strong hydrogen bonds and electrostatic interactions with the brucite-like layers. Displacement of CO_3_^2−^ by HAsO_4_^2−^ requires overcoming a higher energy barrier than the exchange of weakly bound Cl^−^; hence, higher ΔH° values are observed for the LDH–CO3 forms [44,63].

The free energy values, ΔG°, are positive in all considered systems, but this is not evidence of a lack of spontaneity of the adsorption reaction. Rather, it results from the adopted way of expressing the distribution coefficient K_d_ in L/g, which increases the free energy values. The spontaneity of the As(V) adsorption process in this case most likely results from the increase in adsorption values, i.e., the decrease in positive ΔG° values, with increasing temperature. Our intention was mainly to identify differences between the studied sorbents rather than to provide information on the absolute values of changes in thermodynamic functions.

#### 2.2.4. Desorption Studies

The results of the desorption studies of arsenate(V) ions from the four LDH sorbents under investigation, carried out using various eluents, are summarised in Table 9. As(V) desorption was highest in Na_2_CO_3_ and NaOH solutions, and significantly lower in H_2_O and NaCl. For MgAl materials, As(V) recovery was 64–67% in Na_2_CO_3_, 53–65% in NaOH, 14–18% in H_2_O and 17–20% in NaCl. For MgCuZnAl materials, these values were lower, at 42–44%, 38–44%, 2% and 4–5% respectively. This indicates that, under the conditions studied, binary sorbents were more easily regenerable than four-component sorbents.

The effectiveness of the eluents was strongly linked to the chemical conditions of the solution. After desorption, Na_2_CO_3_ maintained alkaline pH_24_ values of around 10.6–10.8, whilst NaOH achieved even higher values of 11.4–11.6. For H_2_O, the final pH was 7.7–7.8, whereas for NaCl it was 6.7–8.1, which explains the low desorption under near-neutral conditions. However, basicity alone does not fully explain the differences between the eluents, as Na_2_CO_3_ was usually as effective or more effective than NaOH, despite a lower final pH. This indicates that its effect stems not only from competition with OH^−^, but also from strong competition between CO_3_^2−^ and arsenate for binding sites and the interlayer space of LDH [18,64].

The influence of the interlayer anion was moderate and dependent on the sorbent composition. For MgCuZnAl, the chloride form yielded slightly higher desorption than the carbonate one for all eluents, whereas for MgAl the trend was not clear-cut: the chloride form desorbed As(V) more readily in Na_2_CO_3_ and NaOH, but in H_2_O and NaCl a slightly higher recovery was obtained for the carbonate form. Higher desorption from MgAl systems in H_2_O and NaCl indicates a greater proportion of a weaker-bound, more reversible As(V) fraction, likely associated with surface interactions. In contrast, the very low desorption from MgCuZnAl-CO3 and MgCuZnAl-Cl in H_2_O (2%) and NaCl (4–5%) suggests that the binding of As(V) in the four-component materials was not solely due to weak electrostatic adsorption.

The lower susceptibility of MgCuZnAl to desorption is consistent with the results of XRD, BET and stability studies, which indicated a more complex and less reversible binding of As(V), particularly in the chloride form. The literature shows that arsenate can be bound to LDH via anion exchange, ligand exchange with layer OH groups, and the formation of inner-sphere surface complexes at the edges of hydrotalcite particles. Additionally, Huang et al. demonstrated that during the adsorption of As(V) onto MgAl-LDH, both interlayer chlorides and carbonates may undergo exchange. Therefore, the lower desorption from four-component materials can most safely be attributed to a higher proportion of the As(V) fraction bound by a combination of interlayer and surface complexation processes, rather than by a single simple mechanism [64,65,66].

The reusability of the investigated LDH sorbents was evaluated in three consecutive adsorption–desorption cycles using NaOH as the desorbing agent (Table 10). Although Na_2_CO_3_ showed the highest As(V) desorption efficiency in the preliminary tests, NaOH was selected for the cyclic experiment to limit the conversion of the chloride forms of LDH into carbonate ones just after the first regeneration cycle. Under these conditions, NaOH removed only part of the adsorbed As(V), but the sorbents still retained the ability to adsorb these anions in subsequent cycles. For all samples, As(V) adsorption decreased markedly after the first cycle, and the desorption efficiency also gradually decreased with repeated regeneration. These results indicate that only a part of the adsorbed As(V) was removed by NaOH under the applied conditions. The cyclic experiment followed the general adsorption trend observed previously. MgCuZnAl-Cl remained the most effective sorbent throughout the test, with adsorption efficiencies of 81%, 37%, and 27% in the first, second, and third cycles, respectively. However, the advantage of the chloride form was not preserved to the same extent for both compositions. MgAl-Cl showed a pronounced decrease after regeneration. The lower desorption values compared with the preliminary desorption experiment may be related to the shorter contact time used in the cyclic tests, which was 1 h instead of 24 h. Therefore, the results of both experiments should not be directly compared as equivalent regeneration conditions, but rather considered as complementary information on the desorption behaviour of As(V)-loaded LDHs. Overall, MgCuZnAl-Cl remained the most effective sorbent in all adsorption cycles, although it did not show the highest desorption efficiency. This indicates that high As(V) uptake was not directly accompanied by easy As(V) release under alkaline regeneration conditions. Thus, the adsorption capacity and regeneration efficiency of the MgCuZnAl materials are not directly proportional. The results confirm the possibility of partial reuse of the studied LDHs, but also show that further optimisation of regeneration conditions is needed before assessing their long-term practical use.

#### 2.2.5. pH-Dependent Stability of the LDH Sorbents

To assess the durability of the sorbents, the effect of the pH of the environment (ranging from 4 to 11) on the dissolution rate of MgAl and MgCuZnAl in their carbonate and chloride forms was investigated. The analysis was made for starting materials, shaken in both water and the As(V) solution, to relate the chemical stability of the materials to the actual sorption conditions. This allowed the influence of the type of interlayer anion, the composition of the layer, and the presence of As(V) on the durability of the tested LDHs to be assessed.

**Figure 11:**
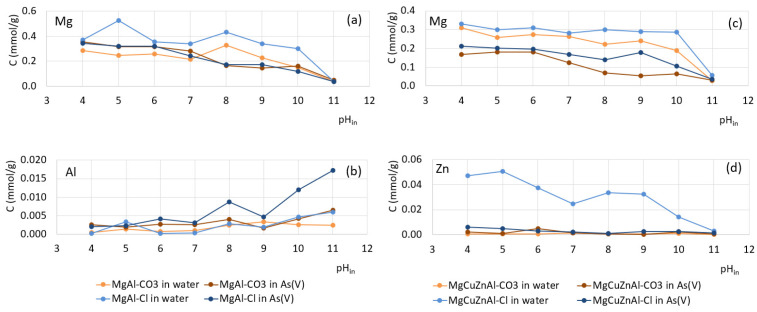
Effect of pH on the release of metal ions from MgAl and MgCuZnAl sorbents in carbonate and chloride forms after shaking in H_2_O or the As(V) solution: (**a**) Mg release from MgAl, (**b**) Al release from MgAl, (**c**) Mg release from MgCuZnAl, and (**d**) Zn release from MgCuZnAl.

Figure 11 shows the relationship between the release of selected metals and the initial pH of the solution (pH_in_) for the systems exhibiting the most pronounced changes. In the case of all sorbents, Mg was the dominant ion released into the solution (Figure 11a,c), which is partly due to its highest content in the brucite-like layers. However, the extent of this predominance was greater than would be expected only from stoichiometry. This observation indicates the incongruent dissolution of the LDH layers with preferential removal of magnesium-containing components, which is consistent with the literature data [38,67]. For each LDH material, strong buffering of the reaction environment was observed. In water, the carbonate forms generated higher final pH values than their corresponding chloride forms: MgCuZnAl-CO3 7.86–9.96, MgCuZnAl-Cl 6.30–7.16, MgAl-CO3 7.52–9.73, MgAl-Cl 6.81–7.59. In turn, after contacting the As(V) solution, these ranges were 7.06–9.85, 6.30–7.72, 7.47–9.76, and 7.12–10.02, respectively. The buffering effect is associated with the presence of CO_3_^2−^ anions in the structure. In the entire pH range, the carbonate forms were generally more stable than the chloride ones, as best evidenced by lower metal leaching and a higher final pH_24h_. For the binary system, the average Mg release was 0.337 mmol/g for MgAl-Cl and 0.218 mmol/g for MgAl-CO3 (Figure 11a), while in the four-component system it was 0.270 and 0.223 mmol/g for MgCuZnAl-Cl and MgCuZnAl-CO3, respectively (Figure 11c). This indicates that the conversion to the chloride form reduces the stability of both materials, while in the four-component system it particularly increases the lability of Zn (Figure 11d). At low pH, the most likely mechanism for the destabilisation of LDH layers is the protonation of the system. In the LDH-CO3 forms, some of the protons are first used for decarbonation of the interlayer, followed by disruption of the hydroxide layer. In the presence of Cl^−^ ions, this buffering step is weaker, so protons destabilise the OH^−^ layer more rapidly, which is consistent with the lower stability of the chloride forms [32,38]. At high pH, the contribution of this mechanism decreases significantly, which is why Mg release decreases. At the same time, however, the amount of dissolved Al increases (Figure 11b), which is explained by its amphoteric nature and the stabilisation of soluble hydroxyl complexes in the alkaline environment [68]. In the case of LDH-Cl, partial exchange of Cl^−^ for OH^−^ and subsequent reorganisation of the structure are also possible.

The presence of arsenate(V) ions altered the stability of the sorbents in a clearly structure-dependent manner, with the strongest protective effect observed for MgCuZnAl-Cl. In this sample contacted with As(V), the average release of Mg decreased from 0.270 to 0.154 mmol/g (Figure 11c), and that of Zn from 0.03 to 0.003 mmol/g (Figure 11d). For MgCuZnAl-CO3, stabilisation concerned mainly Mg (0.223 → 0.108 mmol/g), whereas for MgAl-CO3 it was weak and unsystematic. The observed increase in the sorbent stability can be attributed to the stronger binding effect of As(V) ions, which partially block the solution’s access to the most reactive sites of the network. This is likely related to the involvement of both anion exchange and surface complexation, including the formation of inner-sphere complexes, but without a clear distinction between the contribution of these mechanisms [64,66]. It is worth emphasising during the stability analysis that all studied LDHs strongly buffer the reaction solution. Therefore, it is necessary to correlate metal leaching with the actual final pH_24h_, which allows the impact of the environment itself to be distinguished from the effect of the sorbent structure and the presence of the adsorbate.

Within the environmentally relevant pH range of approximately 6–8.5, typical of arsenic-contaminated groundwater and a significant amount of As-containing wastewater, the release of metals from the investigated LDHs remained at a moderate level and did not indicate any serious limitation to their practical application. MgCuZnAl-Cl performed particularly well, as the presence of As(V) ions additionally stabilised the material under the working conditions. From a practical point of view, this indicates that this sorbent combines a high adsorption capacity with good chemical stability within the most relevant pH range.

### 2.3. Physicochemical Characterisation of LDH Materials After As(V) Sorption

#### 2.3.1. X-Ray Diffraction Analysis (XRD)

**Figure 12:**
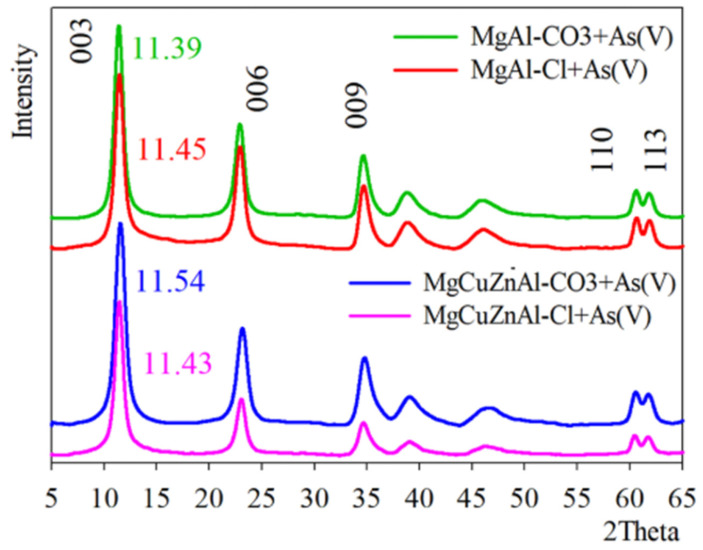
Powder XRD patterns of MgAl and MgCuZnAl in carbonate and chloride forms after As(V) sorption.

The analysis of the XRD spectra following the arsenate sorption process reveals clear differences in both the bimetallic and tetrametallic LDHs, as well as in their carbonate and chloride forms (Figure 12, Table 2). In each case, the layered structure of the material was preserved, and no distinct reflections of new crystalline phases (e.g., metal oxides or crystalline metal arsenates) were observed. The absence of pronounced d(003) shifts confirms the suggestion that the interlayer incorporation of As(V) in LDH-CO_3_, if present, was limited. This is particularly the case for MgAl. Small but consistent shifts in the basal reflection (003) are, in turn, visible for MgAl- and MgCuZnAl-Cl, 11.37° to 11.45° and 11.35° to 11.43°, respectively. The replacement of strongly hydrated chloride anions with higher-charge arsenate ions causes electrostatic interactions between the positively charged hydroxide layer and the anion to become stronger. Consequently, particularly in the case of MgCuZnAl, a more stable hydrotalcite with more intense layer stacking may be formed [8]. An increase in the number of hydrogen bonds can result in the interlayer spacing reduction [42,69]. The absence of clear splitting of the (003) reflection does not indicate formation of two distinct basal populations and is more consistent with the mixed interlayer arrangement. The drying process can also potentially affect the measured basal spacing, as more effective drying can remove more water from the interlayer [32]. As can be seen in the case of layered double hydroxides, it is difficult to unambiguously link the size of the exchanged anion to the change in d(003). Introduction of an anion with a larger ionic radius may result in a decrease in interlayer hydration and an increase in its order, which manifests as a reduction in the basal spacing and an increase in crystallinity.

#### 2.3.2. Surface Area Analysis

Following the sorption of As(V), both carbonate forms exhibited a decrease in the BET surface area and pore volume (Table 3), but the nature of these changes was different. For MgAl-CO3, the BET values decreased from 103 to 65 m^2^/g, the micropore surface area from 16.1 to 8.4 m^2^/g, and the average pore width increased from 12.0 to 16.8 nm, indicating a decrease in the accessibility of the finer fraction of the porous structure. In the case of this material, with an interlayer strongly stabilised by CO_3_^2−^, the most likely mechanism is significant contribution from arsenate bonding on the surface and edges rich in –OH groups, with possible additional stabilisation via hydrogen bonding in the arsenate–water–LDH system [64,66]. In turn, MgCuZnAl-CO3 also exhibited a decrease in the BET surface area (90 → 53 m^2^/g) and the pore volume (0.434 → 0.271 cm^3^/g), but the micropore area increased slightly (6.0 → 7.2 m^2^/g), and the average pore width changed insignificantly (17.5 → 18.2 nm). This suggests a more heterogeneous textural reorganisation. This is most likely due to the combined effects of interlayer sorption and surface complexation, as well as local layer distortions associated with the presence of Cu^2+^ and Zn^2+^ [64,65,70].

The most interesting observations concern both chloride forms after the As(V) sorption. In the case of MgAl-Cl, the BET surface area increased from 20 to 80 m^2^/g, the micropore surface area from 1.7 to 7.2 m^2^/g, and the pore volume from 0.085 to 0.248 cm^3^/g, while the average pore width decreased from 19.7 to 11.4 nm. Such changes indicate a significant reorganisation of the plate aggregates, and consequently an increase in the accessibility of N_2_ to smaller spaces. This may result from the formation or exposure of finer secondary textural porosity. On the contrary, MgCuZnAl-Cl behaved differently, showing a decrease in the BET surface area (59 → 29 m^2^/g) and the micropore area (6.5 → 4.1 m^2^/g), as well as a marked increase in the average pore width (17.8 → 31.2 nm). This indicates a loss of some of the finer porosity and transition to a more coarse texture. The contrast between MgAl-Cl and MgCuZnAl-Cl supports the conclusion that in the binary material, the As(V) sorption is more consistent with the dominant role of electrostatic surface/edge interactions, whereas in the quaternary material, the most likely mechanism involves the combined action of interlayer processes and surface complexation, further modulated by greater layer heterogeneity [64,65,66].

#### 2.3.3. SEM Analysis

SEM images illustrating the surface morphology of the four synthesised LDH samples after adsorption of arsenate(V) ions are shown in Figure 2b,d,f,h. MgAl-CO3+As(V) (b) still retains its plate-like character, but the surface of the aggregates appears more compact and less clearly stratified than before sorption. This corresponds well with the decrease in the BET and micropore surface area. MgAl-Cl+As(V) (d), on the other hand, exhibits the most pronounced reorganisation: the aggregates are more developed, rough and ‘open’, which is consistent with the large increase in the BET surface area, micropore area and pore volume following As(V) sorption. In the case of the four-component material MgCuZnAl-CO3+As(V) (f), the surface becomes more coarse and heterogeneous, but without any significant development of fine porosity. In turn, MgCuZnAl-Cl+As(V) (h) takes the form of larger, compact and thickened aggregates, consistent with a decrease in the BET and micropore area and an increase in the average pore width. Overall, the post-sorption SEM images show that As(V) uptake is accompanied by changes in particle aggregation and surface texture. These changes follow the same material-dependent trend as the adsorption results. In the MgAl system, the preservation of the plate-like morphology and the changes mainly in surface compactness suggest that surface and edge interactions may play a greater role. In contrast, for MgCuZnAl, especially in the chloride form, the higher As(V) uptake is consistent with a more mixed contribution of surface/edge binding, anion exchange, and possible surface complexation [64,66].

#### 2.3.4. FTIR Spectroscopic Analysis

**Figure 13:**
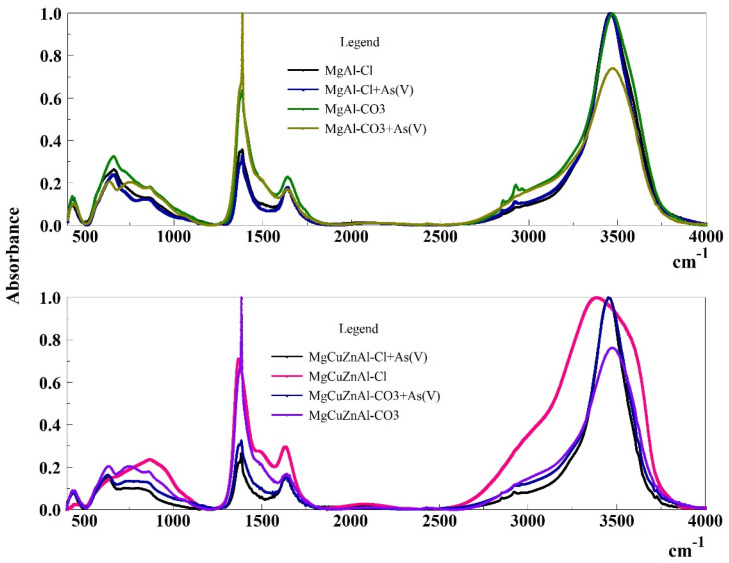
FTIR spectra of the MgAl- and MgCuZnAl-LDH materials before and after As(V) sorption in the range of 400–4000 cm^−1^.

Figure 13 presents the FTIR spectra of the studied LDH materials in the range of 400–4000 cm^−1^. The spectra show characteristic bands assigned to O–H stretching vibrations of hydroxyl groups and interlayer water (3420–3460 cm^−1^), H–O–H bending vibrations (1600–1660 cm^−1^), carbonate vibrations (1360–1370 and 733–735 cm^−1^), weak As–O stretching vibrations in the As(V)-loaded samples (800–900 cm^−1^), and M–O/M–OH lattice vibrations in the low-wavenumber region (500–650 cm^−1^).

##### FTIR Spectra—As–O Vibrational Region (800–950 cm^−1^)

Figure S3 (Supplementary Information) presents the FTIR spectra of the LDH samples in the spectral region corresponding to As–O stretching vibrations. The overlapping FTIR bands in the 800–950 cm^−1^ range were resolved in a Python 3.x environment using the SciPy and NumPy libraries. Before fitting, the spectra were smoothed using a Savitzky–Golay filter with an 11-point window and a third-order polynomial. The baseline was determined using a two-point method and defined as a straight line passing through the points corresponding to the minimum absorbance values in the 800–815 cm^−1^ and 935–950 cm^−1^ ranges. An additional constraint was applied to ensure that the baseline did not exceed the measured absorbance at any point within the analysed range. A sum of pseudo-Voigt functions, expressed as linear combinations of Gaussian and Lorentzian functions, was fitted to the baseline-corrected spectra. The fitting was performed by nonlinear least squares using the scipy.optimize.curve_fit function and the Trust Region Reflective algorithm. The spectra of the samples after As(V) sorption were described using two components: a band located at approximately 829–855 cm^−1^, assigned to As–O stretching vibrations, and a band at approximately 863–874 cm^−1^, associated with LDH lattice vibrations. The spectra of the materials before As(V) sorption were described using a single component assigned to LDH lattice vibrations. The areas of the fitted components were calculated by numerical integration using the trapezoidal rule. The agreement between the fitted models and the experimental data was evaluated using the coefficient of determination, R^2^, which ranged from 0.846 to 0.997. The results of the spectral deconvolution are summarised in Table 11.

The ideal, non-protonated AsO_4_^3−^ ion has a tetrahedral geometry and T_d symmetry, and its antisymmetric stretching vibration ν_3_ is active in the infrared and occurs at approximately 878 cm^−1^ [71]. However, in solutions with a pH corresponding to the experimental conditions, As(V) occurs mainly in the form of HAsO_4_^2−^ with a possible contribution from H_2_AsO_4_^−^ at lower pH values. Protonation and the interaction of arsenate anions with the material’s metal centres result in a reduction in the local symmetry of the AsO_4_ tetrahedron, the lifting of vibrational degeneracy, and the shift and splitting of the As–O bands. The bands identified for the studied materials in the range 829–855 cm^−1^ fall within the range of values described for protonated arsenate ions and arsenates bound to the surfaces of mineral materials [72,73,74], as summarised in Table 12.

##### The Component in the 863–874 cm^−1^ Range

The band at 863–874 cm^−1^ is present in all eight samples (with and without As) and corresponds to vibrations in the brucite layer of LDH: the stretching vibration ν (M–O–M) and the deformation vibration δ (M–OH). The position of this band is stable in all samples, confirming that the structure of the brucite layer is preserved following As(V) adsorption. A slightly broader FWHM in the adsorbed samples (e.g., MgAl-Cl+As(V): 100 cm^−1^ vs. MgAl-Cl: 69 cm^−1^) suggests a slight disruption of lattice regularity following the ‘entry’ of arsenates.

##### The As–O Band (829–855 cm^−1^)—General Interpretation

The component in the 829–855 cm^−1^ range occurs exclusively in the spectra of samples following As(V) sorption; it has therefore been attributed to the stretching vibrations of As–O bonds in arsenate ions bound to LDH materials. Under the pH conditions used in the studies, As(V) was present mainly in the form of HAsO_4_^2−^, with a possible contribution from H_2_AsO_4_^−^. Protonation and the interaction of arsenate ions with metal centres lead to a reduction in the local symmetry of the AsO_4_ tetrahedron, the elimination of vibrational degeneracy, and the shift and splitting of the As–O bands. According to the work by Myneni et al. [71] the ideal, unprotonated AsO_4_^3−^ ion with the symmetry T_d exhibits an antisymmetric stretching vibration ν_3_ at approximately 878 cm^−1^, whilst for the aqueous HAsO_4_^2−^ ion a broad band is observed near 859 cm^−1^, comprising components at approximately 865 and 846 cm^−1^. The positions of the bands determined for the studied materials, ranging from 829 to 855 cm^−1^, are therefore consistent with the range of values reported in the literature for protonated and surface-bound arsenate ions on metal oxides and hydroxides, i.e., approximately 830–865 cm^−1^ (Table 12). The presence of these bands confirms the binding of As(V) by LDH materials and indicates a change in the local environment of the arsenate ions as a result of sorption.

##### As–O Bands in the MgCuZnAl-LDH Materials Containing Cu^2+^ and Zn^2+^

For the MgCuZnAl-Cl+As(V) sample, the component assigned to As–O vibrations was located at 851 cm^−1^ and had an FWHM of 67 cm^−1^. The area of this component, equal to 0.355 a.u., was relatively large compared with the values obtained for the other samples. This result is consistent with the high amount of As(V) bound by the chloride form of the material, as also indicated by the sorption data and XPS analysis. The relatively large width of the As–O component can indicate a heterogeneous local environment of the bound arsenates, resulting from their presence at more than one type of sorption site. The LDH-Cl form enables efficient anion exchange (Cl^−^ → HAsO_4_^2−^), while surface complexation with Cu^2+^ is also possible; consequently, the heterogeneous population of arsenate adsorption sites is found both in the interlayer space (anion exchange) and on the surface (complexation with Cu^2+^). For the MgCuZnAl-CO3+As(V) sample, the As–O component was located at 835 cm^−1^ and had an FWHM of 26 cm^−1^. The strong affinity of CO_3_^2−^ ions for the LDH interlayer limits their exchange with arsenate species. Therefore, interactions occurring at the external surfaces and layer edges can play a greater role in this material. The narrow FWHM of 26 cm^−1^ indicates a more homogeneous population of adsorption sites and may suggest that complexation with Cu^2+^ is the dominant mechanism. The lower position of the band at 835 cm^−1^, compared with 851 cm^−1^ for the chloride form, indicates a different local environment for the As–O bonds, a disruption of AsO_4_ symmetry, or a difference in the interaction between the arsenate species and the surface metal sites.

##### As–O Bands in the MgAl–LDH Materials

In the spectrum of the MgAl-Cl+As(V) sample, the band attributed to As–O vibrations occurs at 829 cm^−1^, which is the lowest wavenumber of all the materials analysed. As this sample does not contain Cu^2+^, the presence of strong surface complexes, such as ≡Al–OH–As or ≡Mg–OH–As, is unlikely [53]. The low position of the band at 829 cm^−1^ should therefore not be interpreted as the result of strong, partially covalent complexation. Instead, it can reflect the specific electrostatic environment of the HAsO_4_^2−^ ions located in the LDH interlayer space. Following the partial exchange of Cl^−^ ions for HAsO_4_^2−^, the arsenate anions are surrounded by positively charged brucite layers containing Mg^2+^ and Al^3+^. Strong electrostatic interactions can lead to a reduction in the local symmetry of the AsO_4_ tetrahedron and a shift in the As–O band towards lower wavenumbers, without the need for the formation of M–O–As covalent bonds. At the same time, due to the significant overlap of the As–O band with the LDH lattice vibration bands, the influence of the deconvolution procedure on the determined position of this component cannot be completely ruled out. In the spectrum of the MgAl-CO3+As(V) sample, on the other hand, the As–O band appears at 855 cm^−1^, which is the highest wavenumber of all the samples analysed. In a binary material containing CO_3_^2−^, HAsO_4_^2−^ ions are not readily introduced into the interlayer space, as the carbonate anions are strongly bound and are difficult to exchange. Furthermore, due to the absence of Cu^2+^, the formation of strong surface complexes involving additional metallic centres is not expected. The As(V) sorption in this material is weaker and is likely to be predominantly electrostatic in nature. The higher position of the As–O band at 855 cm^−1^, closer to the characteristic value for free AsO_4_^3−^ ions of approximately 878 cm^−1^, is consistent with the preservation of the higher tetrahedral symmetry T_d of AsO_4_ in less complexed or electrostatically adsorbed arsenate forms.

To summarise the description of the As–O band positions, the following order can be established: MgAl-Cl+As(V) (829) < MgCuZnAl-CO3+As(V) (835) < MgCuZnAl-Cl+As(V) (851) < MgAl-CO3+As(V) (855) cm^−1^. The observed order of the As–O band positions cannot be interpreted as a simple measure of complexation strength, as the four-component samples containing Cu^2+^ and Zn^2+^, and the two-component MgAl samples, which lack these cations, may bind As(V) via qualitatively different mechanisms. In the MgCuZnAl-CO3+As(V) material, the As–O band appears at 835 cm^−1^. Due to the high stability of CO_3_^2−^ anions in the interlayer space, their exchange for HAsO_4_^2−^ is limited; therefore, surface complexation of As(V) involving Cu^2+^ centres, leading to the formation of Cu–O–As bonds, may play a dominant role. In the case of the MgCuZnAl-Cl+As(V), the As–O band occurs at 851 cm^−1^. The As(V) sorption may occur here both via anion exchange of Cl^−^ for HAsO_4_^2−^ and via surface complexation involving Cu^2+^. The larger FWHM value of this component may reflect the presence of a heterogeneous population of sorption sites and the coexistence of several forms of arsenate binding. For the MgAl-Cl+As(V) sample, the As–O band at 829 cm^−1^ has the lowest position among the analysed materials. In this case, the main mechanism is likely to be the exchange of Cl^−^ ions for HAsO_4_^2−^. The low position of the band may result from strong electrostatic interactions between HAsO_4_^2−^ anions and the positively charged LDH layers, and the associated change in the local symmetry of the AsO_4_ tetrahedron, rather than from the formation of covalent M–O–As bonds.

In contrast, in the MgAl-CO_3_+As(V) material, the As–O band occurs at 855 cm^−1^. Due to the limited exchange of strongly bound CO_3_^2−^ anions and the absence of additional Cu^2+^ centres, As(V) sorption is weaker and is likely to be predominantly electrostatic in nature. The higher position of the band, closer to the value characteristic of free AsO_4_^3−^ ions, may indicate greater preservation of the tetrahedral T_d symmetry of the arsenates and their weaker interaction with the structure of the material.

Based on the analysis, the following conclusions can be drawn:

1. The component in the 829–855 cm^−1^ range was observed only in the samples after As(V) sorption, confirming its association with As–O vibrations in arsenate species bound to the LDH materials. This range is consistent with values reported for protonated and surface-bound As(V) species.
2. The component in the 863–874 cm^−1^ range was present in all samples. The small changes in its position indicate that As(V) sorption did not cause major changes in the local environment of the metal–oxygen bonds. The observed broadening of this component may reflect increased heterogeneity of the local environment.
3. Differences in the width of the As–O band in MgCuZnAl materials
  The As–O band in the MgCuZnAl-CO3+As(V) spectrum is characterised by a small FWHM of 26 cm^−1^, whereas in the MgCuZnAl-Cl+As(V) spectrum it is significantly broader, with an FWHM of 67 cm^−1^. In the four-component carbonate form, a single sorption mechanism is likely to dominate, involving the complexation of As(V) with Cu^2+^ centres, which indicates a relatively homogeneous population of sorption sites. In contrast, in the chloride form, two sorption mechanisms coexist: anion exchange and surface complexation involving Cu^2+^. The presence of different modes of As(V) binding leads to greater heterogeneity in its local environment, which is reflected in the significantly greater width of the As–O band.
4. Interpretation of the position of the As–O band in MgAl-Cl+As(V)
  The position of the As–O band at 829 cm^−1^ in the MgAl-Cl+As(V) spectrum does not indicate surface complexation. The binary MgAl material does not contain Cu^2+^ and is unlikely to form intra-sphere As(V)–Al or As(V)–Mg complexes. The low position of the band may, however, result from an electrostatic disturbance of the T_d tetrahedral symmetry of HAsO_4_^2−^ ions located in the LDH interlayer space. It cannot be ruled out, either, that the value of 829 cm^−1^ is partly due to a deconvolution artefact.

##### The ν(O–H) Vibration Region, 3000–4000 cm^−1^

Figures S4 and S5 (Supplementary Information) show the FTIR spectra of four-component and two-component LDH materials before and after As(V) sorption. The position of the maximum of the broad ν(O–H) band was determined directly from the experimental data as the wavenumber corresponding to the highest absorbance value in the range 3000–4000 cm^−1^. No deconvolution or fitting of model functions was employed in this analysis. The procedure used therefore does not require any assumptions to be made regarding the number and shape of the components constituting the broad ν(O–H) band. The determined positions of the maxima and their shifts following As(V) sorption are summarised in Table 13.

In the case of the four-component chloride-form material, MgCuZnAl-Cl, a distinct shift in the ν(OH) band towards higher wavenumbers by 63.1 cm^−1^ was observed following the sorption of As(V). Weakly interacting Cl^−^ ions do not significantly disrupt the hydrogen-bond network between water molecules in the interlayer space. The introduction of HAsO_4_^2−^ anions via anion exchange, however, can lead to a reorganisation of this network. Strong H_2_O–H_2_O interactions are partially replaced by weaker interactions involving arsenates, resulting in an increase in the O–H vibrational frequency and a shift in the ν(OH) band towards higher wavenumbers. This observation supports the interpretation that anion exchange plays a significant, and probably dominant, role in the sorption of As(V) onto the four-component LDH in its chloride form. In the case of a four-component material in the carbonate form, the direction of the ν(OH) band shift is opposite and amounts to −21.7 cm^−1^. The negative shift may be associated with a surface reaction in which HAsO_4_^2−^ anions replace the hydroxyl groups bound to the surface ≡Cu–OH centres. As a result of this ligand exchange, some of the hydroxyl groups, previously strongly bound to the metal centres, may move to a weaker-bound and freer environment. This leads to a reduction in the average vibrational frequency of the analysed population of OH groups and a shift in the band towards lower wavenumbers. This result indicates a significant, and likely dominant, contribution from surface As(V) complexation in the case of the carbonate form of LDH. In the case of the MgAl materials, no measurable shift in the ν(OH) band was observed following As(V) sorption. For the chloride form, the shift was only +4.3 cm^−1^ and lay within the margin of measurement error, while for the carbonate form, no change in the position of this band was observed. These results indicate that the presence of Cu^2+^ in the structure of four-component LDHs is a significant factor responsible for the large shifts in the ν(OH) band observed following As(V) sorption. In the four-component material MgCuZnAl-Cl, Jahn–Teller distortion in the vicinity of Cu^2+^ ions may lead to the formation of an anisotropic local electrostatic field, which influences the polarisation of neighbouring OH groups in the brucite layers and of interlayer water molecules. The high heterogeneity of the local hydrogen-bonding environments is reflected in the broad ν(OH) band recorded for the MgCuZnAl-Cl prior to As(V) sorption (Figure S4 Supplementary Information). The introduction of HAsO_4_^2−^ anions into the interlayer space as a result of anion exchange may disrupt some of the existing OH–Cl^−^ and OH–OH interactions. At the same time, the presence of tetrahedral arsenate anions may lead to the formation of a more regular and repeatable pattern of interactions with the surrounding OH groups and H_2_O molecules than in the heterogeneous environment present prior to the exchange. The reduction in the diversity of local hydrogen-bonding environments may therefore account for the narrowing of the ν(OH) band observed for the MgCuZnAl-Cl following As(V) sorption. In the case of the MgCuZnAl-CO3, As(V) sorption occurs, as indicated earlier, mainly via surface reactions, whilst arsenate anions do not contribute significantly to the displacement of strongly bound CO_3_^2−^ ions from the interlayer space. The interaction of As(V) with ≡Cu–OH groups occurs primarily on the surface of the material and does not cause a significant reorganisation of the hydrogen-bond network in the interlayer space. As a result, the heterogeneity of the local environment of the OH groups remains similar, and the ν(OH) band width does not change significantly.

#### 2.3.5. XPS Surface Chemical Analysis

The XPS analysis was used to evaluate changes in the surface composition of LDH materials following As(V) sorption and to determine the effect of Cu and Zn addition on the sorption properties of the obtained materials. The survey spectra (Figure S6 Supplementary Information) confirmed the presence of the main components of the hydrotalcite structure, i.e., Mg, Al, and O, as well as Cu and Zn. After the adsorption process, an additional As 3d signal appeared in the spectra, clearly indicating binding of arsenic to the sorbent surface. In the case of the MgAl-LDH sample, the arsenic content on the surface was 1.0 at.% for the carbonate form and 1.2 at.% for the chloride form (Table 14). In the MgCuZnAl samples, the arsenic content increased to 1.3 at.% (carbonate form) and 1.9 at.% (chloride form). The highest amount of adsorbed As(V) was observed for the MgCuZnAl-Cl+As(V) sample, indicating that the addition of Cu and Zn cations into the LDH structure favourably affects the arsenic sorption capacity, especially in the materials containing chlorides in the interlayer space. The maximum position of the As 3d signal (~45.6 eV) corresponds to arsenic in the V oxidation state, indicating that after adsorption, arsenic remained mainly in the form of arsenate(V) [15,75]. Changes in the chloride content were also visible in the analysed samples. For the MgCuZnAl-Cl reference material, the Cl content was 2.7 at.%, while after the arsenic sorption it decreased to 1.1 at.% in the MgCuZnAl-Cl+As(V) sample. A similar trend was observed for the MgAl-LDH samples. This can indicate that chloride ions participated in the As(V) binding process partial anion exchange [65]. The introduction of Cu and Zn into the LDH structure also influenced the relative contribution of other surface components. The MgCuZnAl samples had a lower Mg content and a higher Al content, resulting in a lower Mg/Al ratio compared to the MgAl. This change can be important for the sorption properties of materials, as a higher Al content is associated with an increased positive charge in the LDH layers and a stronger interaction with the anionic arsenic species. The spectra of the MgCuZnAl samples contained Cu 2p3/2 and Zn 2p3/2 signals, confirming the effective incorporation of these metals into the material structure. At the same time, the samples containing Cu and Zn exhibited a higher arsenic content on the surface than their corresponding MgAl materials, suggesting that the presence of these elements favourably affects the As(V) adsorption process [76]. The XPS results confirm that LDH modification with Cu and Zn leads to changes in the material surface chemistry, which translates into better arsenic sorption efficiency.

**Figure 14:**
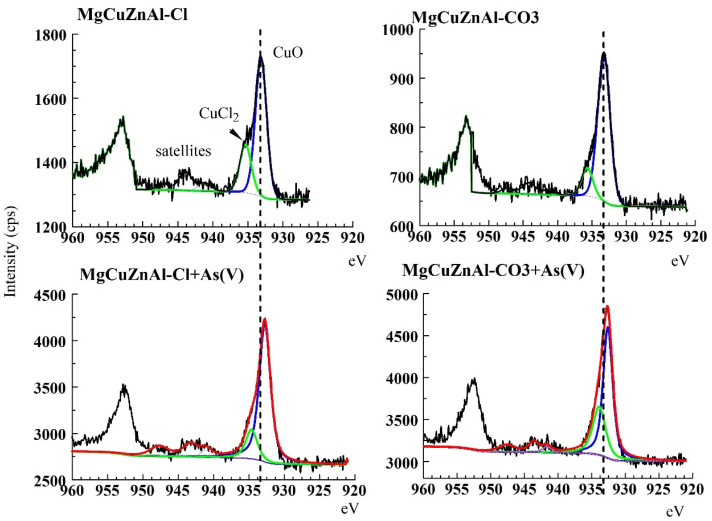
Cu 2p XPS spectra for LDH samples.

**Figure 15:**
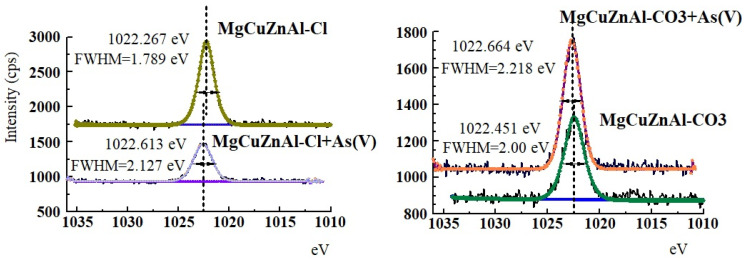
Zn 2p XPS spectra for LDH samples.

**Figure 16:**
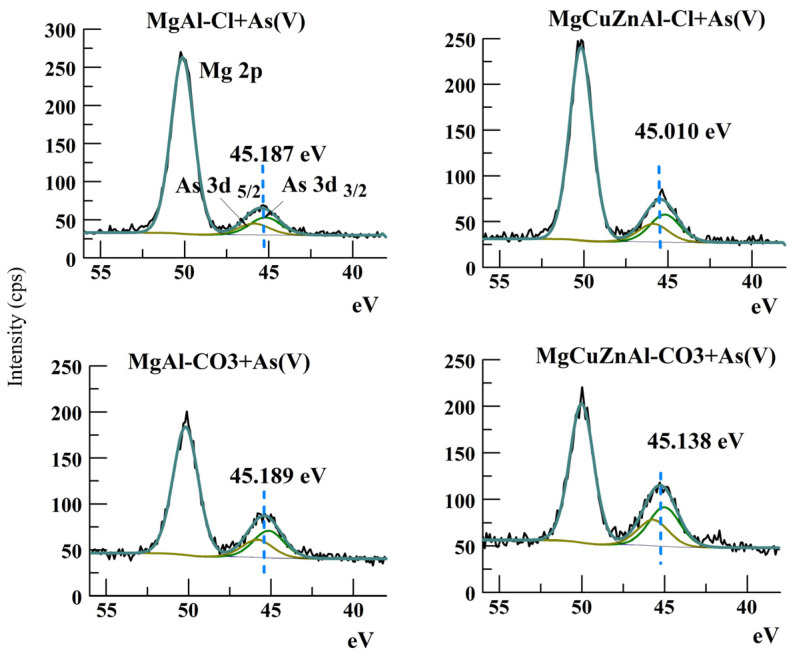
As 3d XPS spectra for LDH samples.

Analysis of high-resolution Cu 2p, Zn 2p and As 3d spectra for MgCuZnAl-LDH materials before and after As(V) sorption (Figure 14, Figure 15 and Figure 16) showed that, following the introduction of As(V) anions, the Cu 2p peak maximum shifted towards lower binding energies for both the chloride and carbonate forms. The opposite trend was observed for the Zn 2p signal, whose peak shifted towards higher binding energies after sorption. The opposite directions of the shifts in the Cu 2p and Zn 2p peaks indicate that the presence of As(V) affects the local electronic environment of Cu^2+^ and Zn^2+^ ions in the LDH layers differently. The Cu 2p3/2 spectrum was analysed considering two main components, attributed to CuO and CuCl_2_ environments [77]. The CuO component can be associated with Cu^2+^ ions present in the vicinity of the OH^−^ groups of the LDH brucite layers, while the CuCl_2_ component relates to an environment with a weaker electron donor, associated with the presence of Cl^−^ ions or axially coordinated ligands in a distorted Cu(II) geometry.

Following the sorption of As(V), a reduction in the energy gap between the CuO and CuCl_2_ components was observed. For the chloride form, this value changed from 2.214 to 2.094 eV, whilst for the carbonate form it changed from 2.415 to 1.592 eV (Table 15). This reduction in the energy gap is therefore evidence that HAsO_4_^2−^ directly modifies the coordination sphere of Cu^2+^, which is a strong argument in favour of the formation of a Cu-As(V) surface complex. The As(V) ion interacts with Cu^2+^ in both types of environment, although with varying strength. At the same time, the contribution of individual components of the Cu 2p3/2 spectrum changed. For the chloride form, this ratio decreased following As(V) sorption from 0.365 to 0.215, which is consistent with the exchange of Cl^−^/HAsO_4_^2−^ in the interlayer space (Table 16). For the carbonate form, the CuCl_2_/CuO ratio increased from 0.158 to 0.491, indicating a more pronounced modification of the surface environment of Cu^2+^. Since carbonate anions are less exchangeable than chloride, surface sorption may be more significant in the carbonate form than the penetration of arsenates into the interlayer space.

The Cu 2p spectra also showed distinct shake-up satellite peaks, characteristic of Cu(II) ions. Their presence indicates that, in the samples analysed, Cu remains in the II oxidation state and is not reduced to Cu(I) [78]. Following the sorption of As(V), all satellite peaks shifted relative to the main CuO peak towards higher binding energy values (Table 15). For the chloride form, these shifts were +0.770, +0.174 and +1.049 eV respectively, while for the carbonate form they were +1.071, +0.223 and +0.914 eV. The values obtained confirm a change in the electronic environment of Cu^2+^ ions following contact between the material and an arsenate solution, which provides further evidence that the Cu–As(V) interaction is of a coordinative nature. The observed shift in the Cu 2p peak, as well as in the satellite peaks, does not necessarily indicate the formation of an inner-sphere Cu^2+^ complex with HAsO_4_^2−^ ions; it may result from a change in the Madelung potential of the entire crystal lattice, which occurs following the addition of As(V) ions into the LDH structure. However, it is highly likely that the As(V) ion acts as an electron donor to Cu^2+^ ions with an unfilled 3d^9^ electron shell. This electron donation reduces the effective charge of the Cu^2+^ nucleus and, as a result, weakens the interaction between the 2p electrons and the Cu^2+^ nucleus, i.e., it lowers the binding energy (BE) of the 2p orbitals. Cu^2+^ ions, as Lewis acids, form exceptionally strong arsenate complexes in aqueous solutions, with stability constants three orders of magnitude higher than those of Zn^2+^ ions with a filled d^10^ electron shell [79]. For this reason, the interaction between Zn^2+^ ions and arsenate ions during the adsorption process is much weaker and contrary to the behaviour of Cu(II) ions. The observed shift in Zn^2+^ towards higher BE values (Figure 14, Figure 15 and Figure 16) is explained by the entry of the highly charged AsO_4_^3−^ into the structure or onto the surface of the LDH, and the increase in the local negative potential in the vicinity of Zn^2+^, which causes electrons to be repelled from Zn^2+^, results in poorer screening of the nucleus and, consequently, a higher BE. This is the Madelung effect acting at a distance, without Zn–As contact. The change in the FWHM value (Table 15) of the chloride form of LDH (increase) for Zn 2p confirms that, in the case of the chloride form of LDH, the As(V) ion enters the interlayer space, reorganising the structure and causing heterogeneity in the environment of the Zn^2+^ ions, whereas in the case of the carbonate form, the As(V) ion adsorbs mainly on the surface, without disturbing the Zn^2+^ environment deep within the structure. The high-resolution As 3d spectra of all samples following As(V) sorption (Figure 16) consisted of a spin–orbital doublet attributed to the As 3d5/2 and As 3d3/2 components. The energy gap between these components was 0.680 eV, and the ratio of the surface areas was consistent with the theoretical value for the As 3d level, resulting from 6:4 degeneracy [80]. The binding energies of the As 3d5/2 component were 45.189 eV for MgAl-CO3+As(V), 45.187 eV for MgAl-Cl+As(V), 45.138 eV for MgCuZnAl-CO3+As(V) and 45.010 eV for MgCuZnAl-Cl+As(V) (Table 17). These values fall within the range characteristic of arsenic at oxidation state V, present in the form of arsenates HAsO_4_^2−^, typically observed at 44.9–45.6 eV [81]. The FWHM values of the As 3d signal ranged from 2.1 to 2.2 eV, and no additional components were observed in the spectra. This indicates the presence of a single dominant form of arsenic on the surface of all samples following As(V) sorption. At the same time, for materials containing Cu and Zn, a slight shift in the As 3d signal towards lower binding energies was observed compared with MgAl materials. This shift may reflect a change in the local electrostatic environment of the arsenates, resulting from the introduction of Cu^2+^ and Zn^2+^ into the LDH layers. However, the exact origin of this effect cannot be reliably determined solely on the basis of the As 3d spectra. The similar FWHM values for both forms of MgCuZnAl indicate, however, that in the four-component materials, the arsenates occur in a similar surface environment, regardless of the type of interlayer anion.

In addition, the surface concentration ratios As/Al, As/Mg, As/Cu and As/Zn, determined on the basis of XPS data, were analysed (Table 14). For the MgAl-Cl+As(V) sample, the As/Al and As/Mg ratios were 0.13 and 0.04 respectively; for MgAl-CO3+As(V) they increased to 0.15 and 0.05. In the case of four-component materials, the differences were more pronounced. For MgCuZnAl-Cl+As(V), the As/Al, As/Mg, As/Cu and As/Zn ratios were 0.08, 0.07, 0.81 and 1.00 respectively; for MgCuZnAl-CO3+As(V) they were 0.11, 0.12, 1.27 and 1.36, respectively. These data indicate that the distribution of arsenic in the layer analysed by XPS depends on both the composition of the LDH layers and the type of interlayer anion.

It should be emphasised that the XPS technique provides information mainly on the composition of the surface and near-surface layers, rather than on the entire volume of the sorbent. The depth of information obtained by XPS is usually a few nanometres, which, in the case of LDH materials, can correspond to several or a dozen or so brucite layers [82]. Therefore, the XPS results should be regarded as characterising the surface and near-surface layers, rather than as a direct reflection of the total amount of As(V) bound throughout the entire mass of the material.

In summary, a detailed analysis of the Cu 2p and Zn 2p spectra confirmed that, following As(V) sorption, the local electronic environment of Cu^2+^ and Zn^2+^ ions in MgCuZnAl-LDH materials changes. The presence of Cu 2p satellite peaks indicates that Cu is retained in the +2 oxidation state, whilst changes in peak positions, FWHM values and the surface ratios As/Al, As/Mg, As/Cu and As/Zn show that the composition and structure of the near-surface layers depend on the type of interlayer anion and the presence of As(V).

### 2.4. Mechanism of As(V) Sorption on LDH Materials

The sorption of As(V) on the LDH materials studied most likely occurs via a mixed mechanism, in which electrostatic interactions, partial anion exchange, surface sorption on hydroxyl groups and, in the case of materials containing Cu^2+^ and Zn^2+^, stronger interactions with additional metallic centres all play a role. The nature of this process depends both on the type of anion present in the interlayer space and on the cationic composition of the LDH layers. The results indicate that the classical MgAl-LDH materials and the four-component MgCuZnAl-LDH bind arsenates in different ways.

In chloride-bearing materials, the partial exchange of Cl^−^ ions for anionic forms of As(V) plays an important role. Cl^−^ ions are less well stabilised in the interlayer space than CO_3_^2−^, and can therefore be more easily replaced by arsenates. Consequently, following sorption, a decrease in chlorine content is observed, along with a slight reorganisation of the interlayer space, as seen in XRD. At the same time, the absence of new crystal reflections indicates that the arsenates do not form a distinct, ordered phase, but are distributed in a dispersed manner, partly in the interlayer space and partly on the surfaces and edges of the layers. In carbonate materials, interlayer exchange is significantly more limited, as CO_3_^2−^ anions exhibit a high affinity for the positively charged LDH layers. In these materials, surface sorption is therefore of greater significance. High values of the K_RP_ parameter for carbonate forms can be interpreted as evidence of the strong affinity of As(V) for surface active sites at low equilibrium concentrations. However, this does not imply effective interlayer exchange; it is more likely that a limited number of strong surface sites are rapidly occupied, after which further increases in sorption are restricted. This conclusion is consistent with the FTIR results. The appearance of the As–O band exclusively following As(V) sorption confirms the binding of arsenates by LDH materials, while its shift relative to the free As(V) ion indicates a reduction in the symmetry following binding to the surface or the interlayer environment. At the same time, the preservation of the LDH lattice bands shows that the underlying layered structure remains intact. Changes in the ν(OH) region, particularly pronounced for MgCuZnAl-Cl, indicate a reorganisation of the environment of the hydroxyl groups and interlayer water, which is consistent with the combination of anion exchange and surface sorption.

The introduction of Cu^2+^ and Zn^2+^ into the LDH structure increases the efficiency of As(V) sorption, but the roles of these ions are not identical. XPS results indicate that, following As(V) sorption, the most pronounced changes occur in the Cu^2+^ environment. Shifts in the Cu 2p signals, changes in the ratio of CuO to CuCl_2_ components, and the presence of satellite peaks characteristic of Cu(II) suggest that Cu^2+^ centres may be involved in stronger interactions with arsenates. This interpretation is consistent with the properties of Cu^2+^ as a Lewis centre and its greater affinity for donor oxygen atoms. The available results support the probable formation of Cu–O–As inner-sphere complexes. The observed changes may also be partly influenced by a change in the local electrostatic field following the introduction of strongly charged arsenate anions. Therefore, the involvement of Cu^2+^ centres and the formation of Cu–O–As inner-sphere complexes should be regarded as a probable and well-founded element of the mechanism, although not as the only process responsible for As(V) uptake. Zn^2+^ appears to play a primarily indirect role. The shift in the Zn 2p signal towards higher binding energy values can be attributed primarily to a change in the local electrostatic environment near Zn^2+^ following the sorption of As(V). Due to its 3d^10^ electron configuration, Zn^2+^ is likely to participate less actively in the direct complexation of arsenates than Cu^2+^. However, it may modify the structure of the LDH layers, increase their heterogeneity and influence the availability and activity of sorption sites.

Pore architecture is also of significant importance. The binary MgAl-LDH is characterised by ink-bottle-type pores, i.e., pores with narrower entrances and wider interiors. Such a geometry may hinder the transport of arsenates to some active sites and promote partial pore blockage. In the MgCuZnAl-LDH materials, the presence of Cu^2+^ and Zn^2+^ leads to a more open and better-connected pore network. This facilitates the diffusion of As(V) anions, improves the accessibility of active sites and reduces mass transport resistance. Therefore, the higher sorption capacity of the four-component materials results not only from changes in surface chemistry, but also from a more favourable pore structure. The kinetics of sorption confirm the multi-stage nature of the process. Initially, As(V) binds rapidly to readily accessible surface sites, accompanied by electrostatic interactions and, in the chloride forms, partial anion exchange. Subsequently, the process proceeds more slowly, which may be attributed to surface reorganisation, diffusion to less accessible sites, and the partial penetration of arsenates into the interlayer space. The good fit of the pseudo-second-order model indicates the involvement of specific surface interactions, while the results of the Weber–Morris model show that intraparticle diffusion is not the sole controlling step. The better fit of the Elovich model for four-component materials, however, supports the conclusion that their surfaces exhibit greater energy heterogeneity. Analysis of pH changes shows that, within the pH range studied, the predominant forms of As(V) are H_2_AsO_4_^−^ and HAsO_4_^2−^, which may participate in both electrostatic interactions and reactions with surface groups. For the MgAl materials, the pH values after sorption remain in the slightly alkaline range, whilst for the MgCuZnAl-Cl a more pronounced decrease in pH is observed. This may indicate a more pronounced reorganisation of the surface and interlayer space, as well as the involvement of more polarised centres associated with the presence of Cu^2+^ and Zn^2+^. The effect of temperature suggests that the sorption process is partly chemical and partly endothermic in nature, particularly in the case of the MgCuZnAl-LDH materials. An increase in temperature may enhance the mobility of arsenates, facilitate access to active sites and promote the reorganisation of the surface and interlayer space.

In summary, the MgAl-LDH binds As(V) mainly through electrostatic interactions and, in the case of the chloride form, partial anion exchange. In the MgCuZnAl-LDH materials, the sorption mechanism is more complex and additionally involves a more favourable pore architecture, greater surface heterogeneity and the possibility of stronger interactions between arsenates and Cu^2+^ centres. The significance of these structural and surface differences is reflected in the sorption capacity values. The maximum adsorption capacities determined for the studied LDHs were as follows: MgCuZnAl-Cl (0.41 mmol/g, 30.72 mg/g) > MgCuZnAl-CO3 (0.37 mmol/g, 27.72 mg/g) > MgAl-Cl (0.30 mmol/g, 22.48 mg/g) > MgAl-CO3 (0.24 mmol/g, 17.98 mg/g). This means that the introduction of Cu^2+^ and Zn^2+^ into the LDH structure and the replacement of the carbonate form with the chloride form increased the As(V) binding capacity by a factor of approximately 1.7. The higher efficiency of the four-component material is therefore not due to a single factor, but to the synergistic effect of easier transport of arsenates within a more open pore network, greater availability of active sites, the presence of additional metal centres and, in the case of the chloride form, the possibility of partial exchange of Cl^−^ anions for arsenates. The obtained values can be compared with the sorption capacities of selected natural and synthetic sorbents described in the literature presented in Table 18. This comparison shows that the LDH materials studied, particularly MgCuZnAl-LDH in the chloride form, exhibit competitive sorption properties towards As(V), whilst offering the possibility of further modification of their composition and surface properties.

## 3. Materials and Methods

### 3.1. Reagents

Al(NO_3_)_3_·9H_2_O, Mg(NO_3_)_2_·6H_2_O, Zn(NO_3_)_2_·6H_2_O, Na_2_CO_3_, and NaOH were purchased from Carl Roth GmbH + Co. KG (Karlsruhe, Germany); Cu(NO_3_)_2_·2.5H_2_O and Na_2_HAsO_4_·6H_2_O were purchased from Sigma-Aldrich, Inc. (St. Louis, MO, USA); NaCl, 36% HCl, 95% H_2_SO_4_, and L(+)-ascorbic acid were purchased from POCH S.A. (Gliwice, Poland); and (NH_4_)_6_Mo_7_O_24_·4H_2_O was purchased from Przedsiębiorstwo Techniczno-Handlowe “CHEMLAND” Mariusz Bartczak (Stargard, Poland). All chemicals were of analytical grade and were used without further purification.

### 3.2. Synthesis of LDHs

The LDH materials Mg_3_Al_1_ and Mg_2_Cu_0.5_Zn_0.5_Al_1_ were synthesised by the co-precipitation method. For Mg_3_Al_1_-CO_3_, the cationic solution (150 mL) composed of appropriate amounts of metal nitrates (Mg(NO_3_)_2_∙6H_2_O and Al(NO_3_)_3_∙9H_2_O) was prepared and added dropwise to the Na_2_CO_3_ solution (1 mol/L, 100 mL) under continuous stirring at a speed of 300 rpm. The pH was maintained at about 10 using NaOH. Then the mixture was aged in a two-stage process (3 h at 80 °C, 24 h at room temperature) using a magnetic stirrer. The suspension was vacuum-filtered using G3 sintered-glass filter funnels, and the precipitate was washed with distilled water approximately five times until neutral pH was reached. After oven drying at 70 °C for 24 h, the resulting solid was carefully ground in an agate mortar. Mg_2_Cu_0.5_Zn_0.5_Al_1_-CO_3_ was obtained in the same way, using a cation solution composed of the corresponding metal nitrates: Mg(NO_3_)_2_∙6H_2_O, Al(NO_3_)_3_∙9H_2_O, Cu(NO_3_)_2_∙3H_2_O, Zn(NO_3_)_2_∙6H_2_O.

Anion exchange was performed by dispersing 1 g of LDH-CO3 in the NaCl solution (2 mol/L, 250 mL), while the pH was maintained at 7 by the addition of dilute hydrochloric acid. The reaction was conducted at room temperature with stirring for 24 h. The final products—Mg_3_Al_1_-Cl and Mg_2_Cu_0.5_Zn_0.5_Al_1_-Cl—were obtained by vacuum filtration followed by oven drying (70 °C, 8 h) and grinding.

### 3.3. Sorption Experiments

Sorption of arsenate(V) ions on the obtained LDH materials was performed in the system containing 0.025 g of the sorbent sample and 25 mL of Na_2_HAsO_4_ solution of a concentration of 0.5 mmol/L, in the case of kinetics (pH = 7.8) and pH-influenced (pH = 4–11) experiments. Adsorption isotherms were determined using As(V) solutions with initial concentrations ranging from 0.1 to 2 mmol/L. All sorption experiments were conducted at room temperature, with the exception of the temperature-effect study, which was performed at 17, 25, 35, and 50 °C. The pH of the solutions before (pH_in_) and after (pH_24h_) a 24 h adsorption process was measured using a PH CHECK digital pH meter (TFA Dostmann GmbH & Co. KG, Wertheim-Reicholzheim, Germany) equipped with automatic temperature compensation. The shaking time was 24 h and a mechanical shaker was used to contact the phases. After the specified time, the systems were filtered and then the concentration of As(V) in the solution was determined by the colorimetric method based on ammonium molybdate [98] and confirmed by the ICP-OES method. Calculations concerning the sorption of arsenate ions were performed based on the following equations:

(19) $$c_{s}=\frac{c_{in}-c_{24h}}{m}\cdot V$$

(20) $$\%A=\frac{c_{in}-c_{24h}}{c_{in}}\cdot 100\%$$

where c_s_ refers to the amount of adsorbed As(V) (mol/g), c_in_ is the concentration of As(V) ions in the initial solution, c_24h_ is the concentration of As(V) ions in the solutions after sorption (mol/L), % A is the percentage of sorption, m is the mass of the corresponding LDH expressed in g, and V is the volume of the solution used for sorption (L).

The LDH+As(V) samples characterised by XRD, FTIR, SEM, BET, and XPS were prepared by saturating the corresponding LDH sorbent in a 10 mmol/L arsenate(V) solution (pH 7.9) for 24 h.

### 3.4. Characterisation Techniques

The quantitative elemental analysis of the solid Mg_3_Al_1_ and Mg_2_Cu_0.5_Zn_0.5_Al_1_ samples, in carbonate and chloride forms, was performed using a Perkin Elmer Optima 7000DV inductively coupled plasma optical emission spectrometer (ICP-OES, Perkin-Elmer, Waltham, MA, USA). A blank sample, 5% HNO_3_ solution, was used. Standard solutions of all analysed metals (Mg, Al, Cu, Zn) were also used (single-element ICP standard, 1000 mg/L, Roth). The powder X-ray diffraction (XRD) analysis (step width of 0.01°, scan speed 5°/min, 5–80° 2θ angle range) on Rigaku MiniFlex II diffractometer (Rigaku Corporation, Tokyo, Japan) using the Cu-Kα radiation (λ = 1.5418 Å) was applied to study crystallinity and phase purity of synthesised LDH samples. The specific surface area was measured by the Brunauer–Emmett–Teller (BET) method under vacuum, for the samples degassed at 120 °C, by N_2_ adsorption–desorption isotherm (at 77 K) using a TriStar II instrument (Micromeritics Instrument Corporation, Norcross, GA, USA). The pore size distribution of the produced material was obtained using the Barrett–Joyner–Halenda (BJH) method. Scanning electron microscopy (SEM) measurements were performed using the Quattro S4 high-resolution scanning electron microscope (Thermo Fisher Scientific Brno s.r.o., Brno, Czech Republic). The samples were prepared by placing powdered material on a carbon tape. All measurements were conducted in the high vacuum mode. Fourier transform infrared spectroscopy in the attenuated total reflection mode (FTIR-ATR) of the obtained materials was performed using a Nicolet 8700A spectrometer (Thermo Electron Scientific Instruments LLC, Madison, WI, USA) equipped with The Smart Orbit diamond attachment. The wavenumber ranged from 4000 to 400 cm^−1^ and the resolution was 4 cm^−1^. The X-ray photoelectron spectroscopy (XPS) studies were carried out using the multi-chamber UHV system (PREVAC). Spectra were collected using the hemispherical Scienta R4000 electron analyser (Gammadata Scienta AB, Uppsala, Sweden). The Scienta SAX-100 X-ray source (Al Kα, 1486.6 eV, 0.8 eV band) equipped with the XM 650 X-Ray Monochromator (0.2 eV band) was used as complementary equipment. The pass energy of the analyser was set to 200 eV for the survey spectra (with 750 meV step), and 50 eV for the regions (high-resolution spectra): C1s, O1s, Zn2p, Al2p, Cr2p and Cu2p (with 100meV step). The base pressure in the analysis chamber was 5 × 10^−9^ mbar. During spectra collection it was not higher than 1 × 10^−8^ mbar. The concentration of Mg, Al, Cu, and Zn ions in the solutions was determined by ICP-OES using the Varian 720-ES spectrometer (Varian Australia Pty Ltd., Mulgrave, Victoria, Australia). Calibration solutions of Al, Cu, Mg, and Zn ions were prepared from the multi-element Certipur^®^ ICP standard solution (10 mg/L). Ultrapure water was obtained using the Polwater DL-2 demineralization system. The room temperature absorption measurements at 430 and 840 nm were performed on the JASCO V-660 UV-Vis spectrophotometer (JASCO Corporation, Hachioji, Tokyo, Japan), using 1 cm quartz cuvettes.

### 3.5. Desorption Studies

MgAl and MgCuZnAl samples in the carbonate and chloride forms, saturated with arsenate(V) ions, were used for desorption experiments. In each experiment, 0.025 g of the sorbent was contacted with 25 mL of the eluent solution. Aqueous solutions of NaCl, NaOH, and Na_2_CO_3_, at a concentration of 10 mmol/L, as well as distilled water, were applied as eluents. The desorption process was performed at room temperature with continuous shaking for 24 h. Next, the suspensions were filtered. The concentration of desorbed As(V) anions in the filtrates was determined using the UV–Vis spectrophotometric method described above. The pH of the eluents was measured before and after the desorption process. Percentage desorption (% D) of arsenate(V) ions was calculated using the following equation:

(21) $$\%D=\frac{n_{aq}}{n_{s}}\cdot 100\%$$

where n_aq_ is the number of moles of As released into the solution during a given desorption step, and n_s_ is the number of moles of As present in the sorbent sample before that desorption step.

The reusability of the studied sorbents was evaluated in three consecutive adsorption–desorption cycles. In each cycle, adsorption was carried out from a 0.5 mmol/L As(V) solution (pH = 7.8) for 1 h. Subsequently, desorption was performed using a 10 mmol/L NaOH solution, with a solid–liquid contact time of 1 h. The As(V) concentration in the filtrates collected after each adsorption and desorption step was determined as described above.

### 3.6. pH-Dependent Sorbent Stability Tests

To evaluate the stability of MgAl and MgCuZnAl sorbents in their carbonate and chloride forms, 0.025 g of each LDH material was shaken with 25 mL of water or the As(V) solution (0.5 mmol/L) at pH in the range of 4–11. The contact time between the phases was 24 h at room temperature. After shaking, the suspensions were filtered, and the resulting solutions were analysed by the ICP-OES technique to determine the concentrations of the released metal ions (Mg, Cu, Zn and Al). As the applied liquid-to-solid ratio was 25 mL per 0.025 g of sorbent, the measured concentrations expressed in mol/L were numerically equivalent to the amounts released per gram of sorbent (mol/g), which enabled direct comparison of the chemical stability of the materials. The pH of the post-reaction solutions was also measured.

All sorption and stability experiments were performed in triplicate for three selected points from each series: the beginning, middle, and end. The values shown in the figures and tables represent the mean of three independent sorption experiments. The experimental deviation did not exceed ±5%.

During the preparation of this manuscript, the authors used the Python programming language, version 3.12.3, using the NumPy (2.4.4), SciPy (1.17.1), and Matplotlib (3.10.8) libraries. Python was used to analyse the FTIR spectra of the studied materials. The spectra were smoothed using a Savitzky–Golay filter (scipy.signal.savgol_filter, window of 9–11 points, 3rd-order polynomial). The baseline was determined using a two-point linear method, constrained so as not to exceed the spectrum value at any point. Component bands were fitted to the baseline-subtracted spectra using pseudo-Voigt functions (a linear combination of Gaussian and Lorentzian functions) via the nonlinear least-squares method (scipy.optimize.curve_fit, Trust Region Reflective algorithm). Band areas were calculated by numerical integration using the trapezoidal rule (scipy.integrate.trapezoid). Fit quality was assessed based on the coefficient of determination (R^2^). In addition, Python was used to evaluate As(V) adsorption kinetics according to the pseudo-first-order, pseudo-second-order, Weber–Morris, and Elovich reaction models. The authors have reviewed and edited the output and take full responsibility for the content of this publication.

## 4. Conclusions

1. The sorption of arsenates onto the chloride and carbonate forms of the MgCuZnAl (Mg_2_Cu_0_._5_Zn_0_._5_Al_1_) and MgAl (Mg_3_Al_1_) occurs via a mixed mechanism involving anion exchange and surface complexation. Anion exchange is of particular significance in the replacement of Cl^−^ ions by HAsO_4_^2−^, while the exchange of CO_3_^2−^ is more limited.
2. The MgCuZnAl sorbents exhibit approximately 30% higher arsenate sorption than the MgAl materials, indicating a greater affinity of four-component LDHs for As(V) ions. The highest sorption capacity, determined using the Redlich–Peterson model, was obtained for MgCuZnAl-Cl (30.72 mg/g), followed by MgCuZnAl-CO3 (27.72 mg/g), MgAl-Cl (22.48 mg/g) and MgAl-CO3 (17.98 mg/g). These results confirm the greater efficiency and affinity of the four-component MgCuZnAl materials for As(V) ions compared with the two-component MgAl materials, with the chloride form exhibiting higher capacity in both groups.
3. The superior sorption properties of the four-component LDH are primarily due to a greater proportion of surface adsorption. This mechanism is more pronounced for the MgCuZnAl-CO3 than for MgCuZnAl-Cl, in which surface complexation coexists with anion exchange.
4. Surface adsorption of arsenates on MgCuZnAl-CO3 and MgCuZnAl-Cl likely involves the formation of surface Cu–O–As interactions. This is facilitated by the d^9^ electron configuration of Cu^2+^ ions and the associated Jahn–Teller distortion, which increases the reactivity of the Cu^2+^ coordination sphere.
5. The contribution of surface adsorption of arsenates is indicated by:
  (a) The different sorption isotherm profiles for the MgCuZnAl-CO3 and MgCuZnAl-Cl, and the higher value of the K_RP_ constant for the carbonate form.
  (b) The sorption kinetics on four-component LDH materials, which are well described by the Elovich model, indicating a significant contribution from chemisorption.
  (c) A shift in the Cu 2p bands in the XPS spectra of the MgCuZnAl-CO3+As(V) and MgCuZnAl-Cl+As(V) samples towards lower binding energies. This change may result from a partial transfer of electron density from the arsenate ions to the Cu^2+^ ions present in the LDH lattice. An increase in electron density in the vicinity of the Cu^2+^ centres enhances the screening of the nuclear charge, leading to a reduction in the binding energy of the Cu 2p electrons. The observed shift therefore indicates a direct interaction between arsenate ions and surface ≡Cu–OH centres. This may indicate the formation of coordination bonds between Cu^2+^ and arsenates, and thus the formation of inner-sphere complexes.
  (d) The presence of the ν(As–O) band at 835 cm^−1^ and its narrow full width at half maximum (FWHM = 26 cm^−1^) for the MgCuZnAl-CO3 indicate a relatively homogeneous bonding of arsenates to surface Cu^2+^ centres. The simultaneous shift in the ν(OH) band by −21.7 cm^−1^ is consistent with the involvement of surface ≡Cu–OH groups in the formation of Cu–O–As complexes. For the MgCuZnAl-Cl, however, the broader As–O band at 851 cm^−1^ indicates the coexistence of surface complexation and anion exchange, whilst the shift in the ν(OH) band by +63.1 cm^−1^ indicates a reorganisation of the hydrogen-bond network in the interlayer space during the exchange of Cl^−^ for HAsO_4_^2−^. Moreover, the difference between the position of the As–O band in LDH samples and that reported for dissolved arsenate species indicates a change in the local environment of As(V), consistent with its binding to the LDH surface.
6. The endothermic nature of arsenate adsorption, i.e., the enthalpy of adsorption ΔH°, is more pronounced in the case of MgCuZnAl sorbents, which can be attributed to the additional energy required for the dehydration of Cu^2+^ ions prior to the formation of a surface complex with arsenate ions.
7. Mathematical modelling showed that HAsO_4_^2−^ and H_2_AsO_4_^−^ ions play the main role in the sorption process, whilst H_3_AsO_4_ and AsO_4_^3−^ practically do not participate in the process.

## Figures and Tables

**Table 1:** Typical pH ranges and arsenic speciation in selected arsenic-bearing wastewaters.

Source/Type of Wastewater	Representative System	Reported pH	Arsenic Speciation Reported in the Study	Predominant As(V) Species at Given pH *	Reported Arsenic Concentration	Reference
Copper smelter wastewater (strongly acidic)	Industrial Cu smelting effluent	~1.4	As(III) dominant	H_3_AsO_4_ (if oxidised to As(V))	3.89 g/L As(III)	[23]
Acidic metallurgical wastewater	Industrial hydrometallurgical stream	1–2	As(III) and As(V)	H_3_AsO_4_/H_2_AsO_4_^−^	~1 g/L total As, 17% As(V) (170 mg/L)	[24]
Acid mine drainage (AMD)	Mining-impacted waters from sulphide ores	Strongly acidic to 3–5	As(III) and/or As(V) (mixed speciation)	H_3_AsO_4_/H_2_AsO_4_^−^	Up to 340 mg/L (mine drainage) and up to 850 mg/L (acid seep)	[25]
As(V)-rich metallurgical wastewater	Real industrial As(V)-rich effluent	~7	As(V) explicitly reported	H_2_AsO_4_^−^/HAsO_4_^2−^	2.8 mg/L As(V)	[26]
GaAs processing wastewater	GaAs industry effluent	6–11	Total As	H_2_AsO_4_^−^/HAsO_4_^2−^	10–2000 mg/L total As (depending on processing step)	[27,28]
Natural groundwater (arsenic-contaminated)	Aquifers (e.g., South Asia, Chile)	6.5–8.5	As(III) and/or As(V) depending on redox	H_2_AsO_4_^−^/HAsO_4_^2−^	<0.5 μg/L up to >5 mg/L; typically <10 μg/L in freshwater	[29]
Gold cyanidation process water	Alkaline leaching circuits	~9	As(III) and As(V)	HAsO_4_^2−^	83.9 mg/L total As	[30]
Alkaline metallurgical wastewater	Smelting/hydrometallurgical alkaline streams	7–12	As(V)	HAsO_4_^2−^/AsO_4_^3−^	50 mg/L As(V)	[31]

* The predominant aqueous species of As(V) are governed by arsenic acid dissociation (pKa_1_ ≈ 2.2, pKa_2_ ≈ 6.8, pKa_3_ ≈ 11.5). Therefore, H_3_AsO_4_ (pH < 2.2); H_2_AsO_4_^−^ (pH 2–7); HAsO_4_^2−^ (pH 7–11); AsO_4_^3−^ (pH > 11) [1,25,29].

**Table 2:** XRD results.

Sample	(003) 2θ	d(003) [Å]	FWHM (003)	(006) 2θ	(009) 2θ	(110) 2θ	d(110) [Å]	(113) 2θ	a = 2·d(110) [Å]	c = 3·d(003) [Å]
MgAl-CO3	11.40	7.76	0.801	22.94	34.61	60.59	1.528	61.84	3.056	23.28
MgAl-Cl	11.37	7.79	0.847	22.76	34.59	60.61	1.528	61.90	3.056	23.37
MgCuZnAl-CO3	11.58	7.64	1.310	23.19	34.83	60.63	1.527	61.81	3.054	22.92
MgCuZnAl-Cl	11.35	7.80	1.125	22.86	34.65	60.46	1.531	61.63	3.062	23.40
MgAl-CO3+As(V)	11.39	7.77	0.928	22.91	34.63	60.60	1.528	61.83	3.056	23.31
MgAl-Cl+As(V)	11.45	7.73	0.964	22.92	34.68	60.64	1.527	61.86	3.054	23.19
MgCuZnAl-CO3+As(V)	11.54	7.67	1.130	23.14	34.76	60.57	1.529	61.77	3.058	23.01
MgCuZnAl-Cl+As(V)	11.43	7.74	0.957	23.04	34.64	60.47	1.531	61.73	3.062	23.22

**Table 3:** Results of the BET analysis.

Sample	BET Surface Area (m^2^/g)	Micropore Area (m^2^/g)	BJH Pore Volume (cm^3^/g)	BJH Average Pore Width (nm)
MgAl-CO3	103	16.1	0.344	12.0
MgAl-Cl	20	1.7	0.085	19.7
MgCuZnAl-CO3	90	6.0	0.434	17.5
MgCuZnAl-Cl	59	6.5	0.255	17.8
MgAl-CO3+As(V)	65	8.4	0.296	16.8
MgAl-Cl+As(V)	80	7.2	0.248	11.4
MgCuZnAl-CO3+As(V)	53	7.2	0.271	18.2
MgCuZnAl-Cl+As(V)	29	4.1	0.230	31.2

**Table 4:** Kinetic model parameters for arsenate sorption on LDH (aqueous phase V = 0.025 L, sorbent mass m = 0.025 g, initial concentration of As(V) in the aqueous phase c_0_ = 0.0005 mol/L, T = 298 K). Standard errors (SE) of linear-regression parameters were calculated from raw kinetic data (t, c_s_(t)) using scipy.stats.linregress (Python 3.12); SE of derived nonlinear parameters (k_2_, α, β) obtained via error propagation/Monte Carlo simulation (20,000 samples).

MgCuZnAl-CO3			
**Model**	**Linearization**	**Parameters (Value ± SE) Unit**	**R^2^**
Pseudo-first-order	ln[c_s_ − c_s_(t)] vs. t	k_1_: (4.4913 × 10^−3^ ± 8.21 × 10^−4^) 1/min	8.3465 × 10^−1^
Pseudo-second-order	t/c_s_(t) vs. t	k_2_: (1.0840 × 10^2^ ± 1.89 × 10^1^) g/(mol·min)	9.9932 × 10^−1^
Weber–Morris	c_s_(t) vs. √t	k_id_: (4.6093 × 10^−6^ ± 1.13 × 10^−6^) mol/(g·√min) C: (1.7479 × 10^−4^ ± 1.86 × 10^−5^) mol/g	7.0328 × 10^−1^
Elovich	c_s_(t) vs. ln(t)	α: 1.9314 × 10^−4^ ± 9.07 × 10^−5^ mol/(g·min) β: (2.6574 × 10^4^ ± 2.21 × 10^3^) g/mol	9.5579 × 10^−1^
MgCuZnAl-Cl			
**Model**	**Linearization**	**Parameters (Value ± SE) Unit**	**R^2^**
Pseudo-first-order	ln[c_s_ − c_s_(t)] vs. t	k_1_: (4.8867 × 10^−3^ ± 7.45 × 10^−4^) 1/min	8.7850 × 10^−1^
Pseudo-second-order	t/c_s_(t) vs. t	k_2_: (9.7599 × 10^1^ ± 1.60 × 10^1^) g/(mol·min)	9.9963 × 10^−1^
Weber–Morris	c_s_(t) vs. √t	k_id_: (5.7029 × 10^−6^ ± 1.46 × 10^−6^) mol/(g·√min) C: (2.7354 × 10^−4^ ± 2.40 × 10^−5^) mol/g	6.8467 × 10^−1^
Elovich	c_s_(t) vs. ln(t)	α: 7.8192 × 10^−4^ ± 5.17 × 10^−4^ mol/(g·min) β: (2.1329 × 10^4^ ± 2.04 × 10^3^) g/mol	9.4355 × 10^−1^
MgAl-CO3			
**Model**	**Linearization**	**Parameters (Value ± SE) Unit**	**R^2^**
Pseudo-first-order	ln[c_s_ − c_s_(t)] vs. t	k_1_: (3.5223 × 10^−3^ ± 1.25 × 10^−3^) 1/min	5.7501 × 10^−1^
Pseudo-second-order	t/c_s_(t) vs. t	k_2_: (3.6729 × 10^2^ ± 1.18 × 10^2^) g/(mol·min)	9.9970 × 10^−1^
Weber–Morris	c_s_(t) vs. √t	k_id_: (1.5294 × 10^−6^ ± 4.26 × 10^−7^) mol/(g·√min) C: (2.1146 × 10^−4^ ± 7.00 × 10^−6^) mol/g	6.4948 × 10^−1^
Elovich	c_s_(t) vs. ln(t)	α: 1.2378 × 10^1^ ± ~2.3 × 10^5^ (undetermined) mol/(g·min) β: (7.9541 × 10^4^ ± 1.10 × 10^4^) g/mol	8.9478 × 10^−1^
MgAl-Cl			
**Model**	**Linearization**	**Parameters (Value ± SE) Unit**	**R^2^**
Pseudo-first-order	ln[c_s_ − c_s_(t)] vs. t	k_1_: (1.4207 × 10^−3^ ± 1.91 × 10^−3^) 1/min	7.1971 × 10^−2^
Pseudo-second-order	t/c_s_(t) vs. t	k_2_: (6.9820 × 10^2^ ± 3.15 × 10^2^) g/(mol·min)	9.9987 × 10^−1^
Weber–Morris	c_s_(t) vs. √t	k_id_: (6.5949 × 10^−7^ ± 3.10 × 10^−7^) mol/(g·√min) C: (2.8725 × 10^−4^ ± 5.09 × 10^−6^) mol/g	3.8381 × 10^−1^

c_s_—pseudo-equilibrium sorption capacity (t = 24 h); c_s_(t)—sorption capacity at time t < 24 h. Note: For MgAl-CO3 and MgAl-Cl, the Elovich α parameter shows an SE comparable to or exceeding its own value, confirming numerically that the Elovich model is poorly constrained for these materials—consistent with the interpretive discussion already present in the manuscript text.

**Table 5:** Parameters of the Redlich–Peterson isotherm for As(V) sorption on LDH.

Sorbent	β	K_RP_ (L/g)	a_RP_ ((L/mol) ^β^)	R^2^	Χ^2^	RMSE (mol/g)
MgCuZnAl-CO3	0.8954	5.009 × 10^2^	6.822 × 10^5^	0.9799	4.53 × 10^−6^	1.17 × 10^−5^
MgCuZnAl-Cl	1.0000	3.805 × 10^2^	9.236 × 10^5^	0.9908	3.39 × 10^−6^	1.24 × 10^−5^
MgAl-CO3	1.0000	2.189 × 10^1^	8.958 × 10^4^	0.9031	1.06 × 10^−5^	1.47 × 10^−5^
MgAl-Cl	1.0000	1.403 × 10^2^	4.744 × 10^5^	0.9819	5.16 × 10^−6^	1.23 × 10^−5^

R^2^—coefficient of determination; χ^2^—chi-square goodness-of-fit statistic; RMSE—root mean square error.

**Table 6:** Influence parameters of individual As(V) species and OH^−^ ions for As(V) sorption on MgCuZnAl-Cl/CO3 and MgAl-Cl/CO3 samples, determined according to Equation (14).

**MgCuZnAl**					
**Influence** **parameter**	**Ion**	**-Cl**	**-CO3**	**Statistical parameters** **-Cl -CO3**	
a	AsO_4_^3−^	−0.36	−1.13	COD: 0.9993 CORR: 0.9996 MSC: 5.9712	COD 0.9979 CORR: 0.999 MSC 4.75
b	HAsO_4_^2−^	−0.10	0.97		
c	H_2_AsO_4_^−^	0.43	3.26		
d	H_3_AsO_4_	0	−3.15		
e	OH^−^	−0.17	−2.8		
**MgAl**					
**Influence** **parameter**	**Ion**	**-Cl**	**-CO3**	**Statistical parameters** **-Cl -CO3**	
a	AsO_4_^3−^	−1.12	−0.65	COD: 0.9939 CORR: 0.9969 MSC: 3.67	COD 0.9918 CORR: 0.9959 MSC 3.38
b	HAsO_4_^2−^	1.84	0.61		
c	H_2_AsO_4_^−^	−0.54	0.27		
d	H_3_AsO_4_	0	−0.16		
e	OH^−^	−0.50	−0.35		

**Table 7:** Thermodynamic parameters calculated for As(V) adsorption on MgAl and MgCuZnAl.

**Linear Fitting Parameters for lnK_eq_ = a(1/T) + b**						
**Sorbent**	**a**		**b**		**R^2^**	
MgAl-CO3	−826		1.6		0.9559	
MgAl-Cl	−230		−0.03		0.8872	
MgCuZnAl-CO3	−1281		3.5		0.9915	
MgCuZnAl-Cl	−531		1.5		0.9901	
**Thermodynamic Parameters Calculated for As(V) Sorption**						
**Sorbent**	**ΔH° [J/mol]**	**ΔS° [J/(mol·K)]**	**ΔG°** **_290_** **K [J/mol]**	**ΔG°** **_298_** **K [J/mol]**	**ΔG°** **_308_** **K [J/mol]**	**ΔG°** **_323_** **K [J/mol]**
MgAl-CO3	6873	13.6	+2980	+2765	+2560	+2534
MgAl-Cl	1917	−0.23	+2029	+1934	+1963	+2020
MgCuZnAl-CO3	10,651	29.2	+2133	+2016	+1579	+1217
MgCuZnAl-Cl	4415	12.8	+688	+628	+516	+268

**Table 8:** Standard hydration enthalpies of selected divalent cations [62].

Ion	ΔhydH° [kJ/mol]	d-Electron Configuration	Comment
Mg^2+^	−1920	d^0^ (no occupied d orbitals)	No LFSE *; regular octahedral coordination sphere
Zn^2+^	−2050	d^10^ (filled d shell)	No LFSE; regular coordination sphere
Cu^2+^	−2100	d^9^ (Jahn–Teller active)	Higher |ΔhydH°| associated with LFSE and distortion of the coordination sphere

* LFSE—Ligand Field Stabilisation Energy.

**Table 9:** Percentage of arsenate(V) ion desorption from carbonate and chloride forms of MgAl and MgCuZnAl using selected desorbing agents (10 mmol/L Na_2_CO_3_, NaCl, NaOH) and distilled water (pH_in_ and pH_24h_ refer to the pH of the solutions before and after desorption, respectively). The phase contact time was 24 h.

Desorbing Agent	pH_in_	MgAl-CO3		MgAl-Cl		MgCuZnAl-CO3		MgCuZnAl-Cl	
		%	pH_24h_	%	pH_24h_	%	pH_24h_	%	pH_24h_
Na_2_CO_3_	10.9	64	10.7	67	10.6	42	10.8	44	10.8
H_2_O	6.7	18	7.7	14	7.7	2	7.7	2	7.8
NaCl	6.8	20	7.9	17	8.1	4	7.2	5	6.7
NaOH	11.6	53	11.5	65	11.4	38	11.6	44	11.6

**Table 10:** Percentage adsorption (% A) and desorption (% D) of arsenate(V) ions for the carbonate and chloride forms of MgAl and MgCuZnAl during three consecutive adsorption–desorption cycles. Adsorption was carried out from a 0.5 mmol/L As(V) solution, while desorption was performed using a 10 mmol/L NaOH solution. The phase contact time for each step was 1 h.

Sorbent	Cycle 1		Cycle 2		Cycle 3	
	% A	% D	% A	% D	% A	% D
MgAl-CO3	43	38	21	33	22	25
MgAl-Cl	52	39	19	30	14	21
MgCuZnAl-CO3	52	31	27	22	23	18
MgCuZnAl-Cl	81	36	37	28	27	22

**Table 11:** Positions (cm^−1^), full widths at half maximum (FWHM, cm^−1^), and areas of the As–O and LDH lattice components obtained by deconvolution of the 800–950 cm^−1^ region.

Sample	As–O (cm^−1^)	As–O FWHM	As–O Area	LDH (cm^−1^)	LDH FWHM	LDH Area	R^2^
**MgCuZnAl-Cl+As(V)**	851	67	0.355	872	40	0.113	0.983
**MgCuZnAl-CO3+As(V)**	835	26	0.099	863	85	0.725	0.996
**MgAl-Cl+As(V)**	829	62	0.166	866	100	1.751	0.997
**MgAl-CO_3_+As(V)**	855	51	0.209	871	44	1.049	0.968
**MgCuZnAl-Cl**	—	—	—	874	168	2.873	0.981
**MgCuZnAl-CO3**	—	—	—	866	46	1.272	0.956
**MgAl-Cl**	—	—	—	870	69	1.125	0.971
**MgAl-CO3**	—	—	—	872	43	1.097	0.846

The designation ‘+As’ in the sample name indicates material following As(V) adsorption; the remaining samples are the starting material without As(V). The LDH network (M–OH, M–O–M) is present in all samples; the As–O component is found exclusively in the samples following adsorption.

**Table 12:** ν(As–O) band positions reported in the literature and a comparison with our own results.

Source	ν(As–O) (cm^−1^)	Citations/Comments
AsO_4_^3−^ in aqueous solution (T_d symmetry)	~878	[71]
HAsO_4_^2−^ (protonated, pH 4–7)	~840–860	Protonation reduces the T_d symmetry and shifts the As–O vibrations towards lower wavenumbers [71].
Zr–O–As on UiO-66 (Zr-MOF, surface complex)	830, 865	New bands at 830 cm^−1^ (Zr–O–As) and 865 cm^−1^ (a combination of symmetric and asymmetric As–O vibrations) following As(V) adsorption [72].
La–O–As on La(OH)_3_ (surface complex)	~800–870	FTIR spectra of lanthanum arsenates in the 800–870 cm^−1^ range [73].
FeAsO_4_ (scorodite)	976, 841, 768, 464	As–O stretching vibrations in the crystal structure of FeAsO_4_ [74].
This work → (MgCuZnAl-Cl+As(V))	851	LDH 4-comp. Both mechanisms: anion exchange + surface complexation with Cu^2+^; wide FWHM 67 cm^−1^.
This work → (MgCuZnAl-CO3+As(V))	835	LDH 4-comp. Complex formation on the surface near Cu^2+^; wide FWHM = 26 cm^−1^.
This work → (MgAl-Cl+As(V))	829	LDH 4-comp. Anion exchange of Cl^−^ by HAsO_4_^2−^; the low band position reflects electrostatic distortion of the T_d symmetry in the interlayer region.
This work → (MgAl-CO3+As(V))	855	LDH 2-comp. Weak electrostatic adsorption; the higher band position indicates greater retention of T_d symmetry.

**Table 13:** Shifts in the ν(OH) band position following As(V) adsorption.

Pair (Without As → +As)	Value Before (cm^−1^)	Value After (cm^−1^)	Δ (cm^−1^)
MgCuZnAl-Cl → MgCuZnAl-Cl+As(V)	3389.3	3452.4	+63.1
MgCuZnAl-CO3 → MgCuZnAl-CO3+As(V)	3474.6	3452.9	−21.7
MgAl-Cl → MgAl-Cl+As(V)	3459.2	3463.5	+4.3
MgAl-CO3 → MgAl-CO3+As(V)	3470.3	3470.3	0.0

**Table 14:** Surface composition and selected atomic ratios of LDH samples determined by XPS.

	MgAl -CO3	MgAl -CO3 +As(V)	MgAl -Cl	MgAl -Cl +As(V)	MgCuZnAl -CO3	MgCuZnAl -CO3 +As(V)	MgCuZnAl -Cl	MgCuZnAl -Cl +As(V)
O (at.%)	47.7	47.4	43.9	48.6	42.8	40.6	39.0	37.5
Mg (at.%)	24.5	24.3	21.7	22.6	17.6	18.2	13.2	16.3
Al (at.%)	7.8	7.8	8.9	7.9	10.1	16.9	8.5	17.1
Cu (at.%)	-	-	-	-	1.1	1.6	0.9	1.1
Zn (at.%)	-	-	-	-	1.2	1.3	1.3	1.4
Cl (at.%)	-	-	4.4	0.7	-	-	2.7	1.1
As (at.%)	-	1.0	-	1.2	-	1.3	-	1.9
Mg/Al	3.14	3.12	2.44	2.86	1.74	1.08	1.55	0.95
As/Cl	-	0.031	-	0.039	-	0.037	-	0.057
As/Al	-	0.15	-	0.13	-	0.11	-	0.08
As/Mg	-	0.05	-	0.04	-	0.12	-	0.07
As/Cu	-	-	-	-	-	1.27	-	0.81
As/Zn	-	-	-	-	-	1.36	-	1.0

**Table 15:** Cu 2p3/2 XPS peak parameters—deconvolution: CuO + CuCl_2_ + satellites.

(**a**) Main peaks and binding energy shifts										
**Sample**		**CuO (Main Peak)**		**CuCl_2_ (Main Peak)**		**Δ(CuCl_2_-CuO) (eV)**	**ΔBE (CuO) (eV)**		**ΔBE (CuCl_2_) (eV)**	
		**BE** **(eV)**	**FWHM (eV)**	**BE** **(eV)**	**FWHM (eV)**					
MgCuZnAl-Cl		933.113	1.897	935.327	1.942	2.214	-			
MgCuZnAl-CO3		933.262	2.099	935.677	1.903	2.415	-			
MgCuZnAl-Cl+As(V)		932.725	2.038	934.819	1.942	2.094	−0.388		−0.508	
MgCuZnAl-CO3+As(V)		932.703	1.875	934.295	2.233	1.592	−0.559		−1.382	
(**b**) Shake-up satellite peaks: binding energies and separations from CuO main peak										
**Sample**	**Satellite 1**				**Satellite 2**		**Satellite 3**			
	**BE (eV)**		**Sat-CuO (eV)**	**Δ(** **Sat-CuO) (eV)**	**BE** **(eV)**	**Sat-CuO (eV)**	**Δ(** **Sat-CuO) (eV)**	**BE (eV)**	**Sat-CuO (eV)**	**Δ(** **Sat-CuO) (eV)**
MgCuZnAl-Cl	941.142		8.029	-	943.918	10.805	-	947.039	13.926	-
MgCuZnAl-CO3	941.381		8.119	-	944.145	10.883	-	947.245	13.983	-
MgCuZnAl-Cl+As(V)	941.524		8.799	+0.770	943.704	10.979	+0.174	947.700	14.975	+1.049
MgCuZnAl-CO3+As(V)	941.893		9.190	+1.071	943.809	11.106	+0.223	947.600	14.897	+0.914

BE = binding energy (eV); FWHM = full width at half maximum (eV); Δ(CuCl_2_-CuO) = peak separation; ΔBE = BE (after) – BE (before); Sat-CuO = satellite BE − CuO BE; Δ(Sat-CuO) = (Sat-CuO) (after) − (Sat-CuO) (before); All Δ values are positive, indicating that all three shake-up satellites shift away from the CuO main peak after As(V) adsorption.

**Table 16:** The percentage contributions of the CuO, CuCl_2_ and shake-up satellite components in the Cu 2p3/2 spectrum, and the CuCl_2_/CuO ratio before and after As(V) adsorption, determined by numerically integrating the fitted peaks using the Gaussian–Lorentzian (GL) mixture-function.

Sample	Main Peaks		Satellite Shake-Up			Sat ∑ (%)	CuCl_2_/CuO
	CuO (%)	CuCl_2_ (%)	Sat 1 (%)	Sat 2 (%)	Sat 3 (%)		
MgCuZnAl-Cl	60.35	22.04	4.51	10.36	2.75	17.62	0.365
MgCuZnAl-CO3	76.50	12.05	2.02	6.85	2.58	11.45	0.158
MgCuZnAl-Cl+As(V)	66.89	14.38	3.48	8.00	7.24	18.73	0.215
MgCuZnAl-CO3+As(V)	58.69	28.79	2.73	4.59	5.20	12.52	0.491

The sum of CuO + CuCl_2_ + Sat ∑ = 100% for each sample; Sat ∑ = the sum of the shares of satellites 1–3.

**Table 17:** XPS binding energies of the As 3d spin–orbit doublet for arsenate-loaded LDH samples; BE centroid = (3 × BE5/2 + 2 × BE3/2)/5.

Sample	As 3d5/2 (eV)	As 3d3/2 (eV)	BE Centroid * (eV)	FWHM (eV)
MgAl-CO3+As(V)	45.189	45.869	45.461	2.200
MgAl-Cl+As(V)	45.187	45.867	45.459	2.108
MgCuZnAl-CO3+As(V)	45.138	45.818	45.410	2.199
MgCuZnAl-Cl+As(V)	45.010	45.690	45.282	2.200

* Weighted average of the spin–orbit doublet components (intensity ratio 3:2).

**Table 18:** Sorption parameters of selected natural and synthetic sorbents for As(V) removal reported in the literature.

Sorbent	Ion Removed	Experimental Conditions	Q_max_, mg/g	Proposed Mechanism	Reference
In-layer sulphur-modified MgAl-LDH	As(V)	Mg/Al ratio 2:1; room temperature (RT); 16 h; pH 9.	40.8	enhanced As(V) binding by S-containing LDH sites	[83]
Fe/Mn–CO_3_-LDHs	As(V)	pH 3; 25–45 °C	40.5–47.0	Freundlich/Langmuir analysis; heterogeneous adsorption	[84]
Chitosan-loaded MgAl-LDH	As	60 °C	69.29	Liu isotherm; surface complexation/electrostatic interactions	[59]
Magnetic alginate–chitosan porous beads	As(V)		14.2 ± 0.4	ligand exchange and electrostatic attraction	[85]
Chitosan–magnetite hydrogel beads	As(V)	pH 7; 25 °C; 36 h	66.89 ± 8.57	Langmuir-type adsorption	[86]
Poly(MIA) ionic-liquid polymer gel	As(V)	pH 8; 25 °C	20.78	ion exchange; pseudo-second-order kinetics	[87]
Fe-BC-500 from mulberry stems	As(V)	pH 4; 25–45 °C	18.51–23.62	Langmuir model; complexation	[88]
Heat-treated cassava chaff	As(V)	equilibrium after approx. 120 min	101	Langmuir model; participation of O-containing groups	[89]
Activated hydrochar from red macroalgae	As(III)/(V)	pH 6	3.83	Langmuir model	[90]
Activated carbon	As(V)	single and competitive As(V)/F^−^ systems	3.58	pseudo-second-order kinetics; Langmuir–Freundlich model	[91]
Zr–FeCl_3_B binary metal oxide	As(V)	pH around 5–6	67.28	Langmuir-type monolayer adsorption	[92]
Porous CeO_2_ with surface hydroxyl groups	As(V)	pH 2.5	56.89	heterogeneous monolayer adsorption; diffusion-controlled steps	[93]
UiO-66-NDC/GO MOF/graphene oxide nanocomposite	As(V)	pH 3	147.06	electrostatic attraction, surface complexation and ion exchange	[94]
Zr-MOF	As(V)	pH 7	278	ligand exchange involving Zr–OH groups	[95]
Mn-MOF-500 carbonised Mn-based MOF	As(V)	288–308 K	126.90–166.10	Langmuir model; chemisorption and pore adsorption	[96]
FAH-rGO/CE-4 Fe–Al hydroxide/rGO composite	As(V)		258.39	high-affinity adsorption on Fe/Al-containing active sites	[97]

## Data Availability

The data supporting the conclusions of this article will be made available by the authors on request.

## Supplementary Materials

The following supporting information can be downloaded at: https://www.mdpi.com/article/10.3390/molecules31152645/s1, Figure S1. N_2_ adsorption–desorption isotherms of MgAl and MgCuZnAl LDH sorbents in carbonate and chloride forms before and after As(V) adsorption.; Figure S2. Hysteresis gap plots for MgAl and MgCuZnAl LDH sorbents in chloride and carbonate forms before and after As(V) adsorption. The hysteresis gap was calculated as the difference between the desorption and adsorption branches of the N_2_ isotherms at the same relative pressure (P/P_0_).; Figure S3. FTIR spectra of: MgAl-Cl/CO3 with and without As(V)); region 800–950 cm^−1^; Figure S4. FTIR spectra of: MgCuZnAl-Cl(with and without As(V); MgCuZnAl-CO3(with and without As(V)); Figure S5. FTIR spectra of: MgAl-Cl(with and without As(V); MgAl-CO3(with and without As(V)); Figure S6. The survey spectrum of the MgCuZnAl-Cl+As(V) sample; Table S1. Quantitative descriptors of the N_2_ adsorption–desorption hysteresis loops for LDH samples before and after As(V) adsorption.

## Abbreviations

The following abbreviations are used in this manuscript:

BET	Brunauer–Emmett–Teller method
BJH	Barrett–Joyner–Halenda method
COD	coefficient of determination
CORR	correlation coefficient
FTIR	Fourier transform infrared spectroscopy
ICP-OES	inductively coupled plasma optical emission spectrometry
LDH	layered double hydroxide
LDH-Cl	chloride form of layered double hydroxide
LDH-CO3	carbonate form of layered double hydroxide
MgAl	simplified notation for Mg_3_Al_1_ layered double hydroxide used in this work
MgAl-Cl	simplified notation for chloride form of Mg_3_Al_1_ layered double hydroxide used in this work
MgAl-CO3	simplified notation for carbonate form of Mg_3_Al_1_ layered double hydroxide used in this work
MgCuZnAl	simplified notation for Mg_2_Cu_0.5_Zn_0.5_Al1 layered double hydroxide used in this work
MgCuZnAl-Cl	simplified notation for chloride form of Mg_2_Cu_0.5_Zn_0.5_Al1 layered double hydroxide used in this work
MgCuZnAl-CO3	simplified notation for carbonate form of Mg_2_Cu_0.5_Zn_0.5_Al1 layered double hydroxide used in this work
MSC	model selection criterion
pH_24h_	pH of the solution after 24 h of reaction
pH_in_	initial pH of the solution
pzc	zero charge point
SEM	scanning electron microscopy
XPS	X-ray photoelectron spectroscopy
XRD	X-ray diffraction analysis
